# Digital versus nondigital behavioral interventions on cardiovascular risk reduction: systematic review and meta-analysis

**DOI:** 10.1093/abm/kaaf043

**Published:** 2025-06-27

**Authors:** Fentaw Tadese Berhe, Desalegn Markos Shifti, J Lennert Veerman, Leopold Aminde, Kedir Yimam Ahmed, Yonatan Moges Mesfin, Kelemu Tilahun Kibret, Habtamu Mellie Bizuayehu, Daniel Bekele Ketema, Daniel Bogale Odo, Subash Thapa, Abel Dadi, Sewunet Admasu Belachew, Meless Gebrie Bore, Zemenu Yohannes Kassa, Abdulbasit Musa Seid, Tahir Ahmed Hassen, Erkihun Amsalu, Teketo Kassaw Tegegne

**Affiliations:** School of Medicine and Dentistry, Griffith University, Gold Coast, Queensland, Australia; Faculty of Medicine, Child Health Research Centre, The University of Queensland, Brisbane, Queensland, Australia; School of Medicine and Dentistry, Griffith University, Gold Coast, Queensland, Australia; School of Medicine and Dentistry, Griffith University, Gold Coast, Queensland, Australia; Rural Health Research Institute, Charles Sturt University, Orange, New South Wales, Australia; Immunity and Global Health, Murdoch Children’s Research Institute, Parkville, Victoria, Australia; Global Centre for Preventive Health and Nutrition, Institute for Health Transformation, School of Health and Social Development, Deakin University, Geelong, Victoria, Australia; First Nations Cancer and Wellbeing Research Program, School of Public Health, The University of Queensland, Brisbane, Queensland, Australia; The George Institute for Global Health, University of New South Wales (UNSW), Sydney, New South Wales, Australia; National Centre for Aboriginal and Torres Strait Islander Wellbeing Research, National Centre for Epidemiology and Population Health, Australian National University, Canberra, Australian Capital Territory, Australia; Rural Health Research Institute, Charles Sturt University, Orange, New South Wales, Australia; Menzies School of Health Research, Charles Darwin University, Darwin, Australia; First Nations Cancer and Wellbeing Research Program, School of Public Health, The University of Queensland, Brisbane, Queensland, Australia; School of Nursing and Midwifery, University of Technology Sydney, Sydney, New South Wales, Australia; School of Nursing and Midwifery, University of Technology Sydney, Sydney, New South Wales, Australia; Australian Living Evidence Collaborations, School of Public Health and Preventive Medicine, Monash University, Melboune, Australia; Centre for Women’s Health Research, College of Health, Medicine and Wellbeing, The University of Newcastle, Newcastle, New South Wales, Australia; Faculty of Medicine and Health, Sydney Medical School, University of Sydney, Sydney, New South Wales, Australia; Institute for Physical Activity and Nutrition, School of Exercise and Nutrition Sciences, Deakin University, Geelong, Victoria, Australia

**Keywords:** behavioral intervention, digital interventions, cardiovascular risk reduction, cardiovascular disease

## Abstract

**Objectives:**

We aimed to assess whether digital behavioral interventions improve cardiovascular risk factors more effectively than nondigital behavioral interventions.

**Methods:**

We searched 7 electronic databases from January 1, 1990, to April 4, 2024. We performed a random-effects meta-analysis to pool the effects of digital versus nondigital interventions on body composition, blood pressure, blood glucose, and lipid concentrations. We also conducted subgroup analyses based on intervention duration, risk of bias, and intervention types. We reported outcomes as mean differences with their 95% confidence intervals (CIs). We assessed the quality of the included studies using the Cochrane Risk of Bias 2 tool.

**Results:**

We included 34 randomized controlled trials with 17 389 participants. The meta-analysis found no significant differences between digital and nondigital behavioral interventions for 11 cardiovascular risk factors. However, subgroup analyses showed that digital dietary interventions significantly reduced body weight (MD = −0.66, 95% CI [−1.26, −0.06]), body mass index—BMI (MD = −0.25, 95% CI [−0.43, −0.07]), and fasting blood glucose (MD = −0.31, 95% CI [−0.57, −0.05]) compared to nondigital interventions. Digital physical activity interventions lowered total cholesterol (MD = −3.55, 95% CI [−4.63, −2.46]) compared to nondigital interventions. Combined digital interventions (dietary, physical activity, and smoking cessation) significantly decreased BMI (MD = −0.20, 95% CI [−0.36, −0.04]) compared to nondigital interventions. No significant differences were found by risk of bias or intervention duration.

**Conclusions:**

Digital behavioral interventions are as effective as nondigital interventions in reducing cardiovascular risk factors, making both essential components of cardiovascular disease prevention and management.

## Introduction

Cardiovascular diseases (CVDs) are a significant global health concern, contributing substantially to morbidity and mortality worldwide.^1^ Nearly 70% of CVD cases and related deaths are attributable to modifiable risk factors. These risk factors include high body mass index (BMI), high fasting plasma glucose, elevated blood pressure, and lipid abnormalities, such as elevated low-density lipoprotein (LDL) cholesterol, reduced high-density lipoprotein (HDL) cholesterol, high triglycerides, and increased total cholesterol.^2,3^

Behavioral interventions, which include modifications in dietary habits, physical activity (PA), and other health behaviors, play a significant role in preventing and managing cardiovascular risk factors such as hypertension, dyslipidemia, obesity, and diabetes mellitus.^4–7^ The importance of multifactorial behavioral interventions in improving health outcomes, quality of life, and reducing healthcare costs is well documented.^7–9^ The World Heart Federation Roadmap emphasizes the necessity of enhancing health literacy, self-management strategies, and national policies to promote healthier behaviors, with a growing emphasis on integrating digital health interventions.^10^

The emergence of digital technologies has transformed healthcare delivery, offering novel platforms for disseminating behavioral interventions and promoting behavior change.^9,11–14^ Digital behavioral interventions are delivered through technologies including smartphone applications, wearable devices, telehealth programs, and online platforms.^15,16^ These interventions offer unique opportunities to improve the accessibility, engagement, and scalability of behavioral modification programs.^17^ These interventions have shown promising results in enhancing healthy behaviors and related clinical outcomes, such as medication adherence.^9^

Despite the increasing adoption of digital health interventions, uncertainty remains regarding their comparative effectiveness against traditional nondigital interventions in mitigating cardiovascular risk factors. Some studies suggest positive outcomes associated with digital interventions,^11,18^ but others highlight possible limitations, including issues with adherence/user engagement and long-term sustainability.^19,20^ Although numerous studies have demonstrated the effectiveness of behavioral interventions, direct comparisons between digital and nondigital approaches remain limited. Therefore, a comprehensive evaluation of the relative efficacy of digital versus nondigital behavioral interventions can help to inform evidence-based decision-making in healthcare policy and clinical practice. Thus, this meta-analysis aimed to compare the effectiveness of digital and nondigital behavioral interventions in reducing cardiovascular risk factors, providing insights to inform evidence-based healthcare strategies.

## Methods

This meta-analysis adhered to the Preferred Reporting Items for Systematic Reviews and Meta-analysis (PRISMA) guideline.^21^

### Search strategy

The Medline, Embase, CINAHL, Cochrane Central Register of Controlled Trials, Web of Science, PsycINFO, and SPORTDiscus with full-text databases were searched from January 1, 1990, to April 4, 2024. The search terms combined 3 key subject areas: digital, behavioral intervention, and cardiovascular risk factors (Supplementary Material 2: Search strategy, pp. 48–68). Manual searches of relevant systematic reviews and reference lists were conducted. Search results were exported to Covidence for duplicate removal, screening, and data extraction.

### Study selection criteria

We included primary studies on behavioral interventions that incorporated at least 1 or more components of PA, dietary habits, alcohol use, smoking cessation, and sleep management. These studies had to be randomized controlled trials (RCTs) involving adults aged ≥18 years, without restrictions based on sex or disease status, and must have directly compared digital and nondigital interventions. Digital behavioral interventions use electronic or online technologies to promote behavior change, such as mobile health apps, web platforms, wearable devices, virtual reality, and social networking tools. These interventions often include features like self-monitoring, goal setting, and tailored feedback. In contrast, nondigital behavioral interventions do not rely on electronic technologies and typically involve face-to-face interactions, such as in-person training, counseling, and health education, or paper-based materials like educational booklets.

The included RCTs had to report 1 or more of the following outcomes: body composition (including body weight, BMI, waist circumference), blood pressure, blood glucose (glycated hemoglobin [HbA1c], fasting blood glucose), or blood lipids (including LDL HDL cholesterol, triglycerides, and total cholesterol).

We excluded studies that did not assess the effectiveness of digital and nondigital behavioral interventions in improving cardiovascular risk factors, as well as other types of studies and publications like systematic reviews, meta-analyses, cohorts, case–control, and cross-sectional studies, conference abstracts, editorials, and letters. Additionally, we excluded RCTs involving pregnant or postpartum women, publications in languages other than English, feasibility or pilot studies, and trials with fewer than 30 participants per arm.

### Screening and data extraction

Two reviewers, F.T.B. and D.M.S., did the screening of titles and abstracts, as well as full-text reviews, using Covidence. The information extracted included study characteristics, participant demographics, and intervention details such as duration of intervention, type of intervention, intervention delivery mode, and outcome measures for body composition, blood glucose, blood pressure, and blood lipids. The outcome data for each study arm were provided as pre- and postintervention mean and standard deviation (SD) or mean change and SD for each study arm. If the data were presented as 95% confidence intervals (CIs), medians, or alternate measures, it was converted to mean and SD for each study arm. Any discrepancies were resolved through consensus or discussion with a third reviewer, T.K.T.

### Risk-of-bias assessment

The risk of bias in individual studies was assessed using the revised Cochrane Risk of Bias Tool (RoB 2)^22^ by 2 independent reviewers: F.T.B. and D.M.S. In the event of any disagreement, a third reviewer (T.K.T.) was consulted to reach a consensus.

### Statistical analysis

We performed a random-effects meta-analysis with the inverse variance method to pool unstandardized between-intervention mean differences, considering between-study heterogeneity. We used the restricted maximum-likelihood estimator to calculate the heterogeneity variance estimator *τ*^2^.^23^ We quantified between-study heterogeneity using the *I*^2^ statistic, which estimates the percentage of the variability in effect estimates that is due to between-study heterogeneity rather than sampling error.^24^

We conducted meta-regression and subgroup analyses to investigate the sources of heterogeneity based on intervention duration, risk of bias, and behavioral intervention type. The duration of interventions was classified according to median duration: The first group consisted of interventions with a duration of ≤26 weeks, while the second group had a duration of >26 weeks. The risk of bias was categorized into 3 groups: low risk of bias, some concerns, and high risk of bias. Types of behavioral interventions include PA, dietary habits, alcohol use, smoking cessation, and sleep management, either alone or in combination. We did not report the meta-regression results, as no statistically significant differences were found for any of the 11 risk factors.

We tested for differences between subgroups using the *Q-test*, with the null hypothesis indicating there is no difference in effect sizes between the subgroups. In meta-analyses involving 10 or more studies, publication bias was evaluated using funnel plots and Egger’s test. Egger’s regression intercept, which assesses the correlation between effect sizes and standard errors, was used to detect funnel plot asymmetry, with a significant association (*P*-value <.05) suggesting potential publication bias.^25^ All analyses were performed using the *metacont* function from *meta* package (version 6.5-0) in R (R Foundation for Statistical Computing; R version 4.2.0).

## Results

### Study selection and characteristics

**Figure 1.**
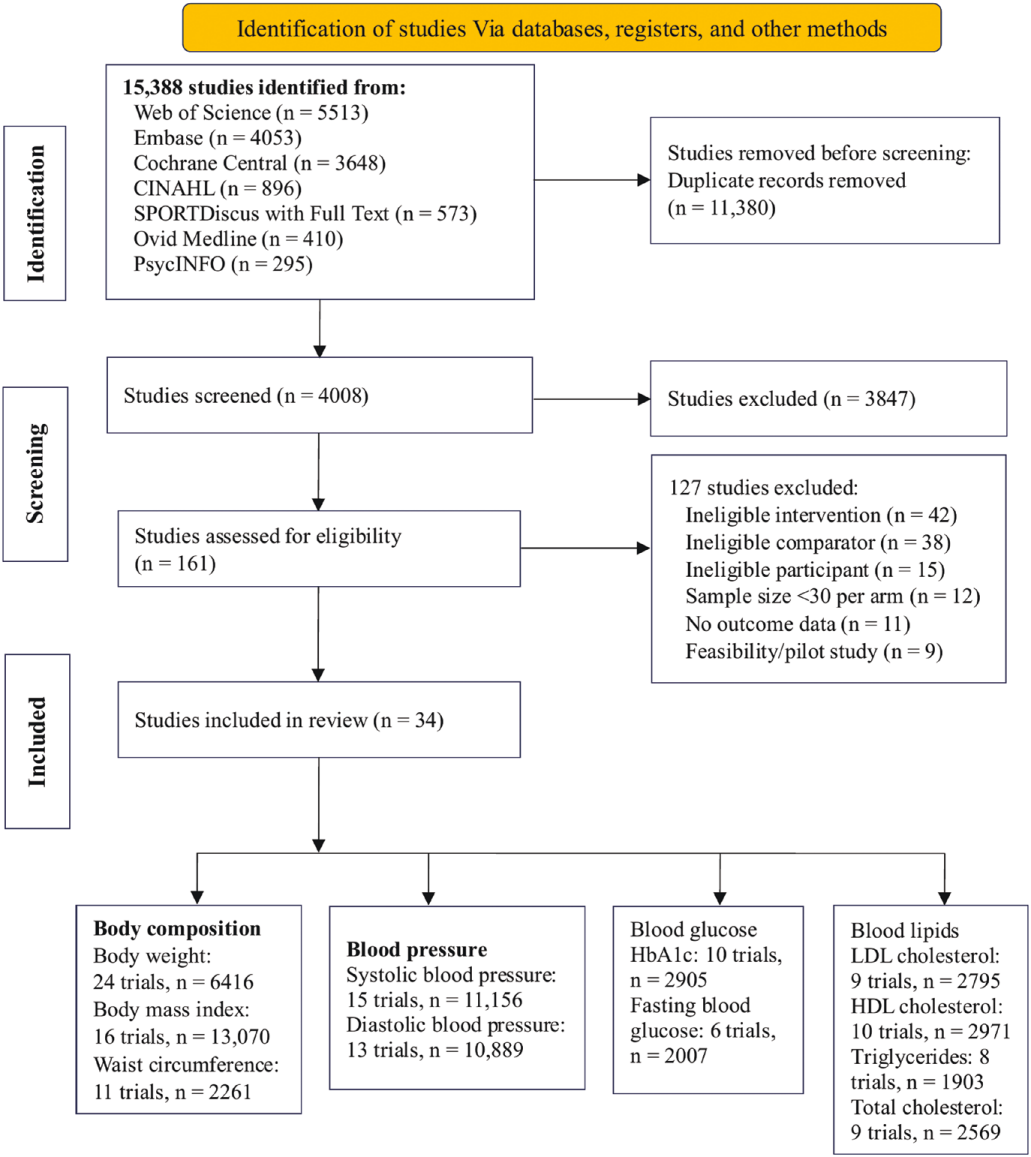
The PRISMA 2020 study selection flow diagram.

A total of 14 681 records were identified, leading to the inclusion of 34 studies^26–59^ with 17 389 participants in this review (Figure 1). Participants comprised 6165 males and 11 224 females, with an average age of 50.22 (SD = 9.50) years. The sample sizes range from 65 to 7610, with a median of 187. Most studies were conducted in the United States (*n* = 23), followed by Spain (*n* = 3) and China (*n* = 2), with the remaining studies (*n* = 6) conducted in Saudi Arabia, Sweden, Bangladesh, South Korea, Germany, and Turkey.

Among the included studies, the majority (*n* = 15, 44.1%) targeted adult individuals aged ≥18 years with cardiometabolic risk factors such as overweight, obesity, prediabetes, metabolic syndrome, or elevated risk for coronary heart disease.^27,28,31,33–35,41,42,44,46,52,54,55,58,59^ A subset of the studies (*n* = 6, 17.6%) enrolled adult participants with chronic conditions, including type 1 or type 2 diabetes mellitus, chronic kidney disease, coronary artery disease, or colorectal polyps.^32,48,49,51,56,57^ A single study was conducted among adult participants with mobility disorders (*n* = 1).^29^ Three studies were conducted in adult female participants, where 1 study was conducted among females with prehypertension,^39^ the second among females with prediabetes,^26^ and the third among female breast cancer survivors with a BMI ≥25 kg/m^2^.^40^ Nine studies (*n* = 9) included healthy adults (≥18 years) with no chronic conditions, or individuals without any self-reported eating disorder.^30,36–38,43,45,47,50,53^

Of the total included studies, the most common behavioral interventions were a combination of PA and diet (PA + D) (*n* = 26), followed by PA alone (*n* = 5), diet (D) alone (*n* = 2), and one that incorporated PA, diet, and smoking cessation (PA + D + Sm). Intervention durations ranged from 6 to 104 weeks, with a median duration of 26 weeks.

The main digital intervention delivery modes included mobile health (mHealth) or telehealth, web-based platforms, wearable devices, virtual reality, and social networking apps, which delivered messages and instructions via text, images, audio, and videos. These tools facilitated self-monitoring, goal setting, and tracking of behaviors, including PA and calorie intake, with regular reminders and tailored feedback to maintain engagement. Web-based platforms offered interactive help queries for expert advice on behaviors, including nutrition and PA. Nondigital interventions are approaches that do not rely on electronic or online technologies, and the delivery modes include face-to-face interactions, such as in-person training, health education, and counseling, as well as paper-based materials, like educational booklets, without in-person or online supervision (Table 1).

**Table 1:** | Study characteristics of included RCTs.

Study description	Delivery methods	
	Digital interventions	Nondigital interventions
Al-Hamdan, 2021^26^ *n* = 83 *n* (sample size) Intervention duration = 26 weeks Intervention types: PA + D	Mobile app and social networking Mobile app-based educational Program about lifestyle modifications The contents include prediabetes, diet, and PA WhatsApp messages were sent to the group by the dietitian and diabetes educator Free consultation	Face-to-face Structured group educational instructions Total of at least 6 sessions covering prediabetes, diet, and PA Paper-based Standard, nonpersonalized lifestyle advice with pamphlets and booklets
Apinaniz, 2019^27^ *n* = 110 Intervention duration = 26 weeks Intervention types: PA + D	Smartphone-installed application Health advice enforced using a Mobile App Program for aerobic exercise and muscle training, a record of food intake, and videos explaining the exercises Text messages for a reminder Messages: one a day during the first month and then two a week until the sixth month	Paper-based: Written health advice Focused on PA and dietary recommendations
Appel, 2011^28^ *n* = 277 Intervention duration = 104 weeks Intervention types: PA + D	Web-based support Motivational interviewing and behavioral self-management Provided learning modules, self-monitoring tools, and feedback Automated re-engagement e-mail messages	Face-to-face: Motivational interviewing and behavioral self-management approaches Weight-loss coaches with Individual and group sessions
Berglind, 2020^29^ *n *= 110 Intervention duration = 12 weeks Intervention types: PA	The App-Based Program Smartphone apps with accelerometers, individually tailored home-based bodyweight exercise Inbuilt goal setting and feedback with face-to-face consultations	Face-to-face (Supervised Health Program) Based on the behavior change model Supervised exercise and health coaching program
Bock, 2019^30^ *n* = 189 Intervention duration = 12 weeks Intervention types: PA	Virtual reality: Exercise video games (EVGs) Changing games during sessions, Gamification features Three times per week, and hospital-based All games were focused on aerobic exercise training	Face-to-face or paper-basedStandard exercise program A center equipped with treadmills and stationary bikes Print materials (weekly) Weekly mailed health and wellness materials
Burke, 2011^31^ *n* = 210 Intervention duration = 104 weeks Intervention types: PA + D	Personal digital assistants (PDAs) Palm PDAs with self-monitoring software Track energy, fat consumption, daily goals, and provide nutrition information PDAs with feedback PDAs with feedback algorithm (tailored daily messages, positive reinforcement, and guidance for goal attainment)	Paper record group Standard paper diaries (record all foods eaten, the calories and fat grams, as well as minutes of exercise) Given a reference book and trained in how to determine portion size
Chamany, 2015^32^ *n* = 738 Intervention duration = 52 weeks Intervention types: PA + D	Tele arm: Phone calls: Participants received four calls Health education, counseling, and behavioral activation by trained behavioral counselors Supervision of health educators through a multidisciplinary team	Paper-based: Print-only intervention Received print self-management materials Mailed every 3 months with mid- and end-of-year phone calls
Clark, 2019^33^ *n *= 99 Intervention duration = 26 weeks Intervention types: PA + D	Internet-based videoconference: Participate in study sessions from their home via videoconference Nutrition and multimodal PA booklet: participants receive “Tip the Calorie Balance” booklets Custom-designed portion control plates with visual cues for vegetables, grains, and proteins Sessions were conducted 2 times per week for 20 weeks and tapered	Enhanced usual care Coaching sessions: weight-related behaviors and assist with problem-solving and action planning by certified coaches in behavior change and fitness Self-monitoring and incentives Specific weight-loss objectives are not provided In-person group: Sessions held at community sites with small groups The same activities with the internet-based group
Ferrara, 2020^34^ *n* = 158 Intervention duration = 52 weeks Intervention types: PA + D	Virtual Lifestyle Management DPP In-person group orientation on using the online platform Applies DPP via online form Streaming audio and interactive visual material Trained coaches used secure messaging to discuss progress, encourage participation, and address questions Behavior change techniques: Included goal setting and self-monitoring through the online platform	Diabetes Prevention Program (DPP), in-person intervention: Uses DPP intervention in worksite Facilitated by trained DPP lifestyle coaches Participants weighed in at the start of each session Self-reported minutes of PA Daily self-monitoring of food intake
Fischer, 2016^35^ *n* = 157 Intervention duration = 52 weeks Intervention types: PA + D	Mobile health Received 6 text messages per week related to the Diabetes Prevention Program (DPP) curriculum Messages included information and encouragement on nutrition, PA, and motivation. Participants were prompted weekly to report their weight Individual motivational interviewing sessions with a coach	Face-to-face (Control group) Received an invitation and access to DPP classes Did not receive weekly text messages or motivational interviewing Eligible for standard-of-care weight-loss resources in a clinical setting
Fottrell, 2019^36^ *n* = 7610 Intervention duration = 61 weeks Intervention types: PA + D + Sm	Mobile Health Intervention: Twice–weekly voice messages Messages were about 1 minute long and had various formats, including minidramas, dialogs, and songs. Informed by behavior change theories and reviewed by medical experts Information on type 2 diabetes signs, symptoms, prevention, care, and risk reduction strategies	Participatory learning and action (PLA) Monthly group meetings A trained facilitator led the group sessions Community mobilization: Community engagement (problem identification, prioritizing, and implementation strategies) Control group: Received usual care Little or no preventative public health campaigning
Gomez-Marcos, 2018^37^ *n* = 833 Intervention duration = 13 weeks Intervention types: PA + D	Mobile Health (Smartphone App) Counseling plus app Records daily portions of food eaten, and PA performed The app generated daily reports and provided personalized recommendations	Face-to-face counseling: Received standardized counseling on PA and diet Counseling sessions were 30 minutes long Provided an informational brochure to reinforce the counseling
Gonzale, 2019^38^ *n *= 833 Intervention duration = 13 weeks Intervention types = PA + D	Mobile Health (Application) Standard counseling plus a Smartphone app Records daily portions of food eaten, and PA performed The app generated daily reports and provided personalized recommendations Adherence to the app was evaluated (recording their data)	Face-to-face: Standardized counseling on PA and nutrition Counseling was adapted based on the participant’s motivation stage Provision of an informational brochure
Hageman, 2014^39^ *n* = 219 Intervention duration = 52 weeks Intervention types = PA + D	Web-Based Intervention Group: Trained and got access to the Wellness for Women: DASHing towards Health website Setting SMART goals for self-monitoring and behavior change Commitment to actions monitored via Telephone call (Goal setting, counseling) Structured telephone script modeled, and the counselor was monitored for quality purposes 18 health promotion newsletters (delivered every 2 weeks for the first 6 months and monthly over the second 6 months) Content-specific information and tailored behavioral messaging It offered a visual feedback display, self-assessment quizzes, and video demonstrations of physical activities	Standard advice-only group: A single 30-minute counseling session with a local extension educator Received printed educational materials and had no further contact with the educator Print-Mailed Intervention Group: Received instruction using paper logs 18 health promotion newsletters (delivered every 2 weeks for the first 6 months and monthly over the second 6 months) Content-specific information and tailored behavioral messaging
Harrigan, 2016^40^ *n* = 67 Intervention duration = 26 weeks Intervention types = PA + D	Telephone weight-loss counseling A lifestyle intervention targeted to reduce caloric intake, increase PA, and provide behavioral therapy Participants received individualized counseling 30 minutes long, once per week (month 1), then every 2 weeks (months 2 and 3), and once per month (months 4, 5, and 6) Record all food and beverage intake, minutes of PA, and pedometer steps (LEAN journal)	Face-to-face counseling: In-person weight-loss counseling Received the same lifestyle intervention with telephone Usual Care Group Participants received brochures linked to clinical usual care
Harvey-Berino, 2010^41^ *n* = 481 Intervention duration = 26 weeks Intervention types = PA + D	Web-based Had access to an online database/website (monitoring as well as educational source) Behavioral change strategies were used Record their daily dietary intake, PA minutes, and weight, and submit weekly Weekly, an hour-long session	Face-to-face (In-person) Behavioral change strategies were used Weekly, an hour-long session Record their daily dietary intake, PA minutes, and weight on paper and submit weekly
Jakicic, 2016^42^ *n* = 470 Intervention duration = 104 weeks Intervention types = PA + D	Technology-enhanced intervention: Short message service (SMS) + Web-based + wearable devices Group-based sessions (Weekly for the first 6 months and monthly from months 7 to 24) Monthly telephone contact and weekly text messages during months 7–24 SMS for prompt engagement and reminders for upcoming sessions Technology-assisted monitoring and reporting	Face-to-face: standard behavioral weight-loss intervention Group-based sessions (weekly for the first 6 months and monthly from months 7 to 24) Monthly telephone contact and weekly text messages during months 7–24 Self-monitored and reported daily intake and PA
Kaur, 2020^43^ *n* = 732 Intervention duration = 26 weeks Intervention types = D	Web-based + SMS + social media SMART Eating website (dietary recommendations, a food measurement guide, BMI calculator, Frequently Asked Questions, and a query box) Multichannel communication Weekly text messages and emails Messages (in the form of text, images, and videos) using social networking (WhatsApp) Interactive query box (questions and responses with an expert)	Face-to-face (Interpersonal) Distribution of a “SMART Eating” kit (A kitchen calendar, dining table mat, measuring spoons) A pictorial pamphlet and visual aids detailing dietary recommendations. Participants were asked to read the pamphlet and share it with their families
Keyserling, 2014^44^ *n* = 336 Intervention duration = 52 weeks Intervention types = PA + D	Web-based 7 counseling sessions: 4 during a 4-month period (monthly, each 45–60 minutes) and 3 for 8 months (each 15–30 minutes at 2-month intervals) 7 counseling sessions using a web program. Personalized risk estimates and chosen risk-reducing strategy: Educational content prepared on web pages. Interactive web program Subsequent visits are monitored by the web. Resource provision (Heart to Health cookbook, pedometers, PA logs)	Face-to-face (counselor-delivered) The sequence and educational content are the same Personalized risk estimates and chosen risk-reducing strategy: content-specific education. Subsequent visits monitored by telephone. Resource provision (Heart to Health cookbook, pedometers, PA logs)
Kim, 2010^45^ *n* = 1114 Intervention duration = 26 weeks Intervention types = PA + D	Self-help (SH) plus counseling (SH + C) group The same printed materials were given to participants randomized to the SH group. Individually tailored structured telephone counseling sessions (a total of 9; 6 main and 3 booster)	Paper-based: Self-help (SH) group Receive only the self-help print materials. Received 3 books (grocery lists, recipes, visual guides for portion control, and increasing PA) Tips on rewards, motivation, planning for challenges, goal tracking, and managing negative thoughts
Krukowski, 2011^46^ *n* = 318 Intervention duration = 26 weeks Intervention types = PA + D	Web-based Weekly virtual group sessions conducted via synchronous “chat” on a secure website. 24 sessions; each session lasted 60 minutes. Offered the same behavioral weight control program content. Daily self-monitoring through paper	Face-to-face: In-person group sessions lasted for 60 minutes and occurred weekly 24 sessions; each session lasted 60 minutes. Offered the same behavioral weight control content. Online diary, daily self-monitoring
Lachausse, 2012^47^ *n* = 176 Intervention duration = 12 weeks Intervention types = PA + D	Internet-based My Student Body (MSB) Interactive, Internet-based program to provide nutrition and physical fitness education. Instructed to visit the MSB Nutrition (via a link on a secure Web) For at least 2 hours per week over 12 weeks	Face-to-face: On-campus course Once a week for approximately 2 hours for 12 weeks In-person MSB nutrition
Lee, 2019^48^ *n* = 65 Intervention duration = 13 weeks Intervention types = PA + D	Mobile Health + SMS: Smartphone Application Usage Provides health information to the user, evaluating PA with a pedometer. Monthly telephone interview	Paper-based: a diary to note exercise and food intake. Mailed health-related newsletters
Li, 2022^49^ *n* = 100 Intervention duration = 6 weeks Intervention types = PA	Supervision Group (SG): WeChat Home-based online-supervised exercise program (HOSEP) Structured exercise sessions that were supervised by a cardiac rehabilitation (CR) physical therapist	Face-to-face: Conventional health education Home exercise program booklet delivered. Online supervision or structured exercise sessions provided to the SG
Luley, 2014^50^ *n *= 118 Intervention duration = 52 weeks Intervention types = PA + D	4 Sigma Telephone Coaching (4S) Group Provided instructions and recommendations on diet and PA Provided with accelerometers (Aipermotion 440) Received monthly telephone calls from specialist doctors and nurses who provided feedback and motivation based on their telemonitored data	Active Body Control (ABC) The same advice was given Provided with accelerometers Weekly personalized feedback and motivational information based on their telemonitored data Control group: Received instructions and recommendations on diet and PA
Lutes, 2017^51^ *n* = 200 Intervention duration = 52 weeks Intervention types: PA + D	Small changes intervention group with a pedometer Received a 16-week EMPOWER treatment manual, recording forms, a weight scale, a glucose monitor, and a pedometer. Supported by Community Health Workers (CHWs) Session topics included self-monitoring, goal setting, nutrition, and PA	Mail-based education group Received educational materials regarding diet, medications, monitoring blood glucose, and engaging in PA 16 mailings
McDoniel, 2010^52^ *n* = 111 Intervention duration = 12 weeks Intervention types = D	Web-based Received a nutrition plan based on their measured resting metabolic rate (RMR) using a validated hand-held indirect calorimetry device. Individual nutrition plans were developed using software. Self-monitoring of food intake, activity, and body weight through software Automated emails and newsletters	Face-to-face and paper-based Standard Nutrition Plan A standard 30-day paper journal tracks daily nutrition, PA, and body weight. Individual counseling using motivational interviewing
Patrick, 2009^53^ *n* = 65 Intervention duration = 16 weeks Intervention types = PA + D	SMS: Personalized, nonrepetitive SMS and MMS messages Dietary recommendations, PA, and behavioral strategies Sent 2–5 times daily Database for storing records and messages Tools for technical monitoring to detect issues and alert the case manager to ensure user satisfaction Brief monthly phone calls from a health counselor	Paper-based 1–2 pages of print materials once a month for 4 months Tips on nutrition, PA, and weight loss
Sherwood, 2006^54^ *n* = 666 Intervention duration = 104 weeks Intervention types = PA + D	Mobile Health: Phone intervention (a series of telephone calls) 10 sequential interactive lessons with feedback after each session Topics included nutrition, PA, and behavior change techniques Program expectations were outlined in an introductory call Scheduled sessions for discussing progress and setting goals.	Paper-based Mail intervention with 10 sequential interactive lessons with feedback after each session Mailed a manual and feedback form Topics included nutrition, PA, and behavior change techniques Progress, setting goals and actions, and feedback via mail
Shuger, 2011^55^ *n* = 98 Intervention duration = 39 weeks Intervention types = PA + D	Armband alone group (SWA-alone): SenseWear, web-based Personalized web account Real-time feedback on energy expenditure, PA, and daily steps Instructed to wear 16 hours a day, 7 days a week Combined GWL and SWA group (GWL + SWA) Received all components of the GWL and SenseWear™ platform	Group-based behavioral weight loss education group (GWL) Received 14 GWL sessions facilitated by a trainer Based on Active Living Every Day (ALED) and Healthy Eating Every Day (HEED) Standard Care (control group) Received a self-directed weight-loss manual Manual aimed to promote a healthful diet and PA Cognitive and behavioral strategies were used Additional 6 one-on-one telephone counseling
St-Jules, 2023^56^ *n* = 185 Intervention duration = 26 weeks Intervention types = PA + D	Social Cognitive Theory-based Behavioral Group Counseling iPads pre-loaded with WebEx for videoconferencing Group-based videoconferencing based on intensive behavior therapy for obesity Weekly session for the first 4 weeks, then biweekly up to 20 weeks Structured presentation containing educational and behavioral elements Technology-based self-monitoring (Monitoring) iPads pre-loaded with WebEx for videoconferencing Group-based videoconferencing Structured presentation containing educational elements Weekly session for the first 4 weeks, then biweekly up to 20 weeks Used smartphone App (Diet and PA) and aligned with the group sessions’ educational material Personalized feedback reports via e-mail Combined: The interaction of SCT and Monitoring	Advice Group Received written intervention targets by mail Follow-up calls with a registered dietitian Mailed monthly 1-page educational handouts
Terkes, 2023^57^ Sample size = 65 Intervention duration = 6 weeks Intervention types: PA	Intervention group Online-supervised exercise program via video conferencing Expert help with audiovisual support Exercises (warm-up, aerobic, strength training, yoga, and balance)	Control group Unsupervised exercise training group Informed by using the lecturing method The same exercise with the intervention group
Thomas 2019^58^ *n* = 220 Intervention duration = 78 weeks Intervention types = PA + D	Smartphone-based behavioral obesity treatment Delivered via a study application for smartphone devices 5-minute skills training videos delivered 3 times weekly for 6 months, then twice–weekly for 6 months, and weekly for the final 6 months Daily self-monitoring using the MyFitnessPal app Electronically generated feedback The App lets you set goals, get reminders, and report reminders Experience sharing	Group-based behavioral obesity treatment Group sessions facilitated by experts Weekly for 6 months, biweekly for the following 6 months, and monthly for the final 6 months Behavioral skills were applied (stimulus control, meal planning, problem-solving) Daily self-monitoring with tools like paper diaries, nutritional reference book Feedback: praise, suggestions, and encouragement
Wong 2023^59^ Sample size = 176 Intervention duration = 24 weeks Intervention types = PA	Mobile Health LIP delivered via the MetS mobile application (MetS app) View the educational booklet on their smartphones Daily automated encouraging health messages to maintain interest and adherence Self-monitoring, exercise goal setting, and recording Alerts with advice for abnormal health measures Congratulatory remarks for achieving the exercise goal, A reminder if not logging in for 2 weeks	Paper-based Lifestyle intervention program (LIP) via booklet The booklet consists of the facts about MetS and advice on diet, exercise, medication, lifestyle, and stress management

Abbreviations: D, Dietary; mHealth, Mobile Health; PA, physical activity; SMS, short message service.

### Risk-of-bias assessment

**Figure 2.**
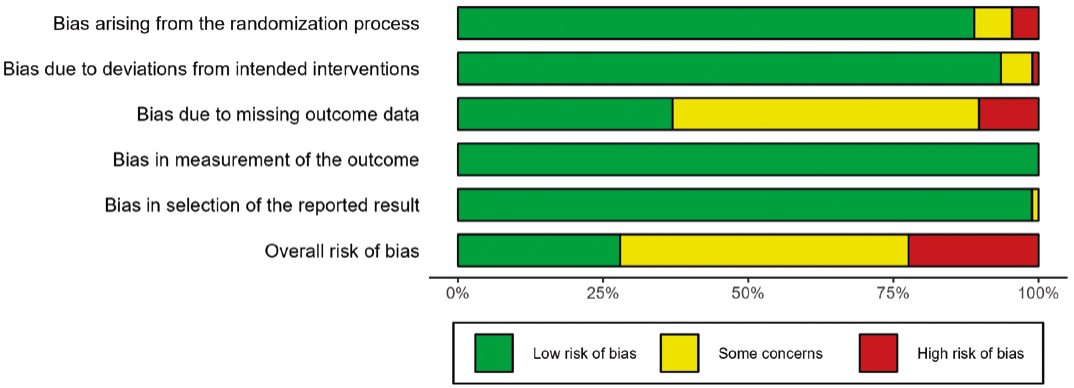
Cochrane risk of bias for the included studies.

Overall, of the 34 RCTs, 21 studies were judged as having either a high risk of bias (*n* = 13) or some concerns (*n* = 8) (Figure 2). Bias due to missing outcome data was the most common source of risk (*n* = 2 high risk, *n* = 10 some concern), followed by bias due to the randomization process (*n* = 4 high risk, *n* = 6 some concern).

### Overall intervention effects

**Figure 3.**
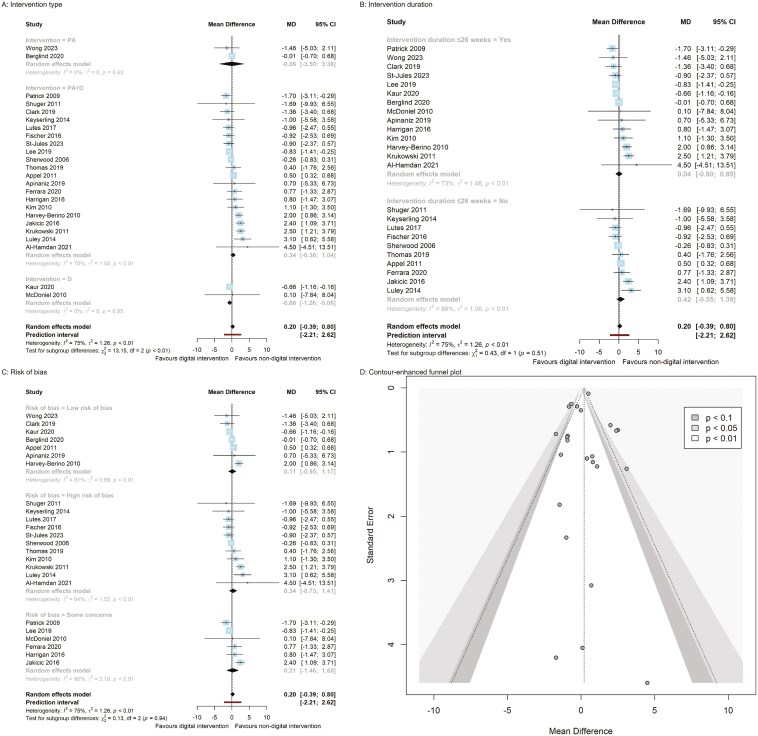
Body weight panel plot (A–C: Subgroup analysis forest plots; D: funnel plot).

**Figure 4.**
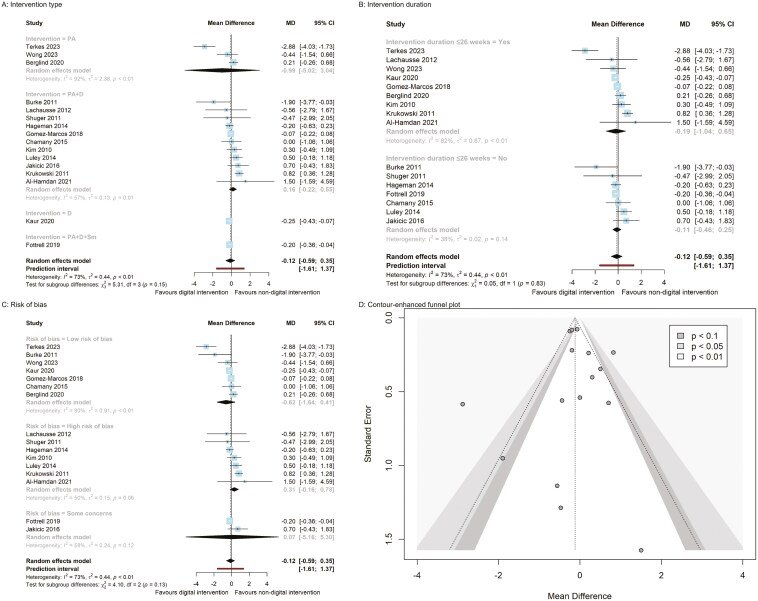
BMI panel plot (A–C: Subgroup analysis forest plots; D: funnel plot).

**Figure 5.**
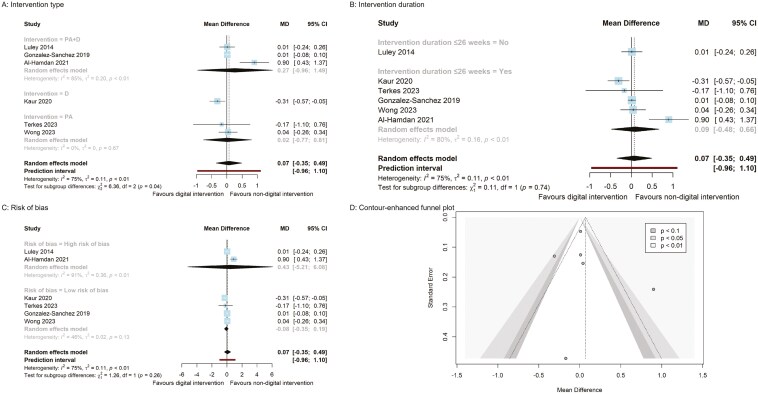
Fasting blood glucose panel plot (A–C: Subgroup analysis forest plots; D: funnel plot).

**Figure 6.**
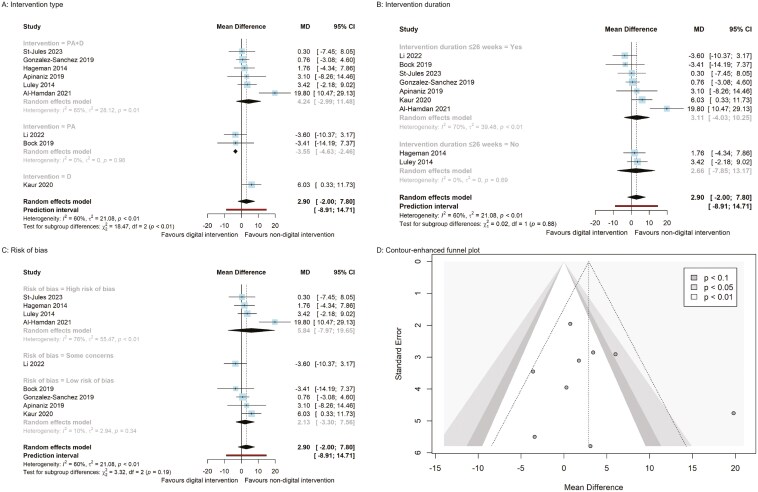
Total cholesterol panel plot (A–C: Subgroup analysis forest plots; D: funnel plot).

The overall pooled effect size of digital behavioral interventions (see Figures 3–6, pp. 45–48) showed no statistically significant differences across all 11 cardiovascular risk factors (body weight, BMI, waist circumference, systolic and diastolic blood pressure, fasting blood glucose, HbA1C, LDL and HDL cholesterol, triglycerides, and total cholesterol) compared to nondigital behavioral interventions. Significant heterogeneity was observed for most pooled estimates (*I*^2^ ≥ 55%, *τ*^2^ ≥ 0.44, *P* < .01), except for waist circumference, LDL and HDL cholesterol (*I*^2^ ≤ 41%, *τ*^2^ ≤ 1.44, *P* ≥ .08), where no heterogeneity was observed (see Figures 3–6, pp. 45–48).

### Subgroup analyses

The subgroup analyses of behavioral interventions revealed greater effects on body weight, BMI, fasting blood glucose, and total cholesterol. Specifically, digital dietary intervention compared to nondigital dietary intervention demonstrated a significantly greater reduction in body weight (MD = −0.66, 95% CI [−1.26, −0.06] kg) based on 2 RCTs (Figure 3, p. 45). Both digital dietary (MD = −0.25, 95% CI [−0.43, −0.07]) and PA + D + Sm interventions (−0.20, 95% CI [−0.36, −0.04]) resulted in BMI reduction compared to nondigital interventions, though each estimate originated from a single study (Figure 4, p. 46). A digital dietary intervention subgroup from 1 RCT demonstrated a significant decrease in fasting blood glucose levels (MD = −0.31, 95% CI [−0.57, −0.05] mmol/L) compared to nondigital dietary intervention (Figure 5, p. 47).

Additionally, the pooled estimate from 2 RCTs on PA (MD = −3.55, 95% CI [−4.63, −2.46] mg/dL) indicated a significant reduction in total cholesterol compared to nondigital PA intervention (Figure 6, p. 48). Conversely, a digital dietary intervention from a single RCT (MD = 6.03, 95% CI [0.33, 11.73] mg/dL) resulted in a significant increase in total cholesterol compared to a nondigital dietary intervention (Figure 6). However, there were no significant differences between digital and nondigital behavior interventions when considering the risk of bias and the duration of the interventions (Figures 3-6 and pp. 45–48).

### Publication bias

Publication bias was not detected by Egger’s regression intercept for all outcomes: body weight, BMI, waist circumference, systolic blood pressure, diastolic blood pressure, HbA1C, and HDL cholesterol. Additionally, the funnel plots did not show asymmetry, indicating that there was no significant publication bias (see Figures 3–6, pp. 45–48).

## Discussion

This systematic review and meta-analysis aimed to compare the effectiveness of digital and nondigital behavioral interventions in reducing cardiovascular risk factors. Although the overall results showed no statistically significant differences in effect between digital and nondigital interventions, the subgroup analyses revealed significant differences. Digital dietary interventions had a significantly greater effect in reducing body weight, BMI, and fasting blood glucose levels. Similarly, digital PA interventions significantly lowered total cholesterol, whereas digital PA + diet + smoking (PA + D + Sm) interventions significantly reduced BMI compared to nondigital interventions. Those who showed statistically significant differences between digital and nondigital interventions had effect sizes that fell short of established clinical significance thresholds for most cardiovascular risk factors; however, they still possess clinical relevance, highlighting the potential for significant health improvements. These differences could be attributed to the features of digital interventions, which often employ multichannel communication approaches, including technology-assisted supervised exercise programs. Such features may enhance engagement and adherence, positively influence exercise and dietary behavior, and potentially contribute to the observed effects on cardiovascular risk factors.^43,60–62^ Thus, it is important to consider the evolving nature of digital technologies and their potential impact on cardiovascular risk reduction.

The findings of this study indicate that digital and nondigital behavioral interventions have comparable effects on various cardiovascular risk factors, including body composition, blood pressure, blood glucose, and blood lipids. Studies show that both approaches hold promise in promoting cardiovascular health among diverse populations.^8,11,60,63^ However, digital behavioral interventions offer unique advantages that can enhance accessibility, engagement, and scalability compared to traditional nondigital interventions.^13,64–66^ The integration of smartphone applications, wearable devices, telehealth programs, and online platforms provides individuals with convenient and personalized tools to monitor and manage their health behaviors effectively.^64^ For instance, exercise prescriptions alongside focused behavioral counseling can be efficiently managed through digital interventions. Additionally, digital interventions can potentially reach a broader audience, including individuals with limited access to traditional healthcare services or those residing in remote areas.^13,17,60,66^ Furthermore, digital behavioral interventions can also be cost-effective, which is a considerable advantage over nondigital interventions.^67^

One of the key strengths of digital interventions is their ability to deliver tailored and interactive content, enhancing user engagement and motivation.^17,64^ By leveraging features such as real-time feedback, goal setting, and social support networks, digital platforms can empower individuals to make sustainable behavioral changes and adhere to treatment regimens.^61,65^ The flexibility of digital interventions allows for continuous monitoring and adjustment of interventions based on individual preferences and progress, thereby optimizing intervention effectiveness over time.^61^ Moreover, digital interventions offer opportunities for data-driven decision-making and personalized interventions through advanced analytics and machine learning algorithms.^61^ By analyzing user-generated data, digital platforms can identify patterns, trends, and predictors of cardiovascular risk, enabling healthcare providers to deliver targeted interventions tailored to individual needs.^61^ Despite these advantages, digital health interventions face challenges, particularly in engaging diverse populations, such as individuals with low digital literacy, those with low socioeconomic status, those living in remote areas, and those with limited tracking participation, as sustained engagement can be difficult.^64,68^ Conversely, conventional face-to-face interventions offer distinct advantages, such as behavioral counseling, prompt feedback, positive rapport, and the use of verbal, nonverbal, and contextual cues that promote interpersonal connections and facilitate behavioral change.^69,70^ Despite these benefits, they often come with higher costs for staffing and facilities, challenges with accessibility and scalability, and variability in outcomes influenced by facilitator experience.^70,71^ Considering these strengths and limitations, along with the varied needs across different settings and contexts, implementation strategies that integrate digital and nondigital interventions hold promise for optimizing their effectiveness in reducing cardiovascular risk factors.

While this meta-analysis provides valuable insights, several limitations should be acknowledged. First, subgroup analyses revealing significant differences were based on a limited number of trials for certain outcomes, necessitating cautious interpretation of the findings and indicating the need for larger studies within each subgroup to validate these findings. In addition, none of the included studies reported body composition indices, such as the waist–hip ratio (WHR). Thus, future studies should consider and assess the effect of behavioral interventions on anthropometric measures of abdominal obesity, including the WHR, as these measures are suggested to be superior to BMI in predicting cardiovascular risk.^72,73^ Second, significant unexplained heterogeneity was observed among subgroups, suggesting variability in intervention effects across studies. This highlights the importance of identifying and addressing sources of heterogeneity through more robust study designs and standardized methodologies. Future research should explore innovative approaches to optimize the effectiveness of digital interventions, such as incorporating gamification elements, virtual reality experiences, and artificial intelligence algorithms. Longitudinal studies are needed to evaluate the long-term sustainability, scalability, and cost-effectiveness of digital interventions in real-world settings.

Third, the meta-analysis did not exclude populations based on disease condition, which may have resulted in a heterogeneous sample where intervention effects could vary significantly between individuals with different health statuses (eg, CVD vs non-CVD, prediabetes vs diabetes). Fourth, the analysis did not account for certain intervention characteristics, such as intensity, delivery modes (eg, SMS vs web-based, in-person vs paper-based), as well as mechanisms of change or the presence of additional interventions like medication adherence, which could further influence the effectiveness of behavioral interventions. Moreover, varying intervention durations across included studies may introduce variability in outcomes and limit comparability. Focusing on specific intervention types, durations, or standardized follow-up periods in future primary studies and meta-analyses could provide more precise estimates of intervention effects over time.

Fifth, methodological limitations, such as a high risk of bias in some studies, may have influenced the validity and reliability of the findings. Prioritizing high-quality RCTs with rigorous methodological standards is essential to minimize bias and enhance the reliability of the evidence in future research efforts. Finally, despite efforts to assess publication bias, its presence cannot be entirely ruled out. Including unpublished studies or conducting sensitivity analyses could help mitigate potential biases and provide a more comprehensive understanding of the effects of digital and nondigital behavioral interventions on cardiovascular risk reduction.

## Conclusion

Digital behavioral interventions are as effective as nondigital interventions in reducing cardiovascular risk factors, making both essential components of CVD prevention and management.

## Supplementary Material

kaaf043_suppl_Supplementary_Materials_1-8

## Data Availability

All data generated or analyzed during this study are included in this published article and its supplementary information files.

## Transparency statements

*Study registration:* This study was not registered.*Analytic plan registration:* The analysis plan was not pre-registered.*Availability of data:* The studies included in this systematic review and meta-analysis are publicly available. The data extraction tables used in the analysis are available upon request from the corresponding author.*Availability of analytic code:* The analytic code is not available in a public archive but may be shared upon request by contacting the corresponding author.*Materials availability:* all materials used to conduct the study are not publicly available; however, the detailed search strategy is provided as a  supplementary file.

For each of the 5 transparency practices, you will provide a statement that accurately describes your article. The following examples may be adapted for use. If none of the statements accurately describe your article, please provide a statement. **Statements should be provided on the cover page and will be published with your manuscript.**

1. **Study registration**. Study registration involves declaring the study design, variables, and treatment conditions in an independent, institutional repository (eg, http://clinicaltrials.gov, https://osf.io/, https://www.socialscienceregistry.org/). A study is considered pre-registered if it is registered in a repository prior to the study began. Consider the following examples:

◦ The study was pre-registered at ________________________________ *(insert name of repository and link to study registration).*

◦ This study was registered after the study began. The study is registered at ___________ (*insert name of repository and link to study registration*).

◦**This study was not formally registered.**☒

2. Analytic plan pre-registration. Pre-registration of an analytic plan involves specification of the planned sequence of analyses or the statistical model that will be reported. Reporting the planned outcome variables alone does not qualify as analytic plan pre-registration. Consider the following examples:

◦ The analysis plan was registered prior to beginning data collection at ____________ (*insert name of repository and link to study registration*).

◦☒ **The analysis plan was not formally pre-registered.**

3. Data availability: Indicate whether the data underlying the presented analyses will be made available in a third-party archive/repository. If data is available in an archive, indicate if this is fully available to the public or has protected access (available for limited audience, with clearly defined requirements and steps to obtain data). If data is not in a public archive, describe any steps needed to access the data. Consider the following examples:

◦ De-identified data from this study are available in a public archive: __________ *(provide URL).*

◦ De-identified data from this study are available in a protected archive: ____________ (*provide URL*). Data can be obtained by _____________ (*briefly describe steps and requirements to access data*)

◦☒ **De-identified data from this study are not available in a public archive. De-identified data from this study will be made available (as allowable according to institutional IRB standards) by emailing the corresponding author.**

4. Analytic code availability. Analytic code refers to the programming code used to conduct the analyses in the statistical software. Consider the following examples:

◦ Analytic code used to conduct the analyses presented in this study are available in a public archive: ______________ *(provide URL)*

◦☒**Analytic code used to conduct the analyses presented in this study are not available in a public archive. They may be available by emailing the corresponding author.**

◦ There is not analytic code associated with this study.

5. Materials availability. Materials include any stimuli used in this study, intervention protocols, and survey instruments and items. Consider the following examples:

◦ All materials used to conduct a study are available in a public archive: ____________ (*provide URL*).

◦ Some of the materials used to conduct the study are presented in a public archive: ____________ (*provide URL*).

◦☒**Materials used to conduct the study are not publicly available.**
