# Supplementary material for: Digital versus nondigital behavioral interventions on cardiovascular risk reduction: systematic review and meta-analysis
Source: Ann Behav Med. 2025 Jun 27;59(1):kaaf043. doi: 10.1093/abm/kaaf043 (PMC12203340; doi:10.1093/abm/kaaf043)
Supplement: kaaf043_suppl_Supplementary_Materials_1-8 [file kaaf043_suppl_supplementary_materials_1-8.docx]

**Electronic Supplementary Materials (ESM)**

Supplementary Material 1: Search strategy

OVID MEDLINE Search

Date of search: 04/04/2024

| Digital interventions | Lifestyle | CVD risk factor | RCT |
| --- | --- | --- | --- |
| Telemedicine/ OR Telephone/ OR Videoconferencing/ OR Wearable Electronic Devices/ OR Video Games/ OR Virtual Reality/ OR Internet-Based Intervention/ OR Mobile Applications/ OR Cell Phone/ OR Smartphone/ OR Computers, Handheld/ OR Text Messaging/ OR (telemed* OR tele-med* OR telehealth* OR tele-health*).ti,ab. OR (ehealth* OR e-health* OR electronic health* OR mhealth* OR m-health* OR mobile health*).ti,ab. OR (wearable* adj3 (devic* OR technolog* OR sens*)).ti,ab. OR ((digital* OR remote OR internet* OR online* OR on-line* OR social media* OR virtual* OR web-based*) adj3 (coach* OR health* OR interven* OR program* OR therap*)).ti,ab. OR (health* adj3 (chatbot* OR technolog* OR websit*)).ti,ab. OR (text messag* OR text* OR SMS OR multi-media messag* OR multimedia messag* OR MMS OR intsnat messag* OR picture messag* OR audio messag*).ti,ab. OR Gamification/ OR (wii fit OR online forum* OR on-line forum* OR discussion board*).ti,ab. | Health Behavior/ OR Health Promotion/ OR Risk Reduction Behavior/ OR Behavior Therapy/ OR Cognitive Behavioral Therapy/ OR Motivation/ OR Health Education/ OR Life Style/ OR Healthy Lifestyle/ OR ((lifestyle OR life style OR behavio?r) adj3 (chang* OR modif* OR program* OR therap* OR intervent*)).ti,ab. OR (health habit* OR unhealthy lifestyle*).ti,ab. OR  Exercise Therapy/ OR Exercise/ OR Physical Fitness/ OR Sports/ OR Walking/ OR Running/ OR Swimming/ OR ((physical OR cardio* OR aerobic OR fitness OR endurance OR flexibility OR strength OR resistance) adj3 (activit* OR train* OR exercise*)).ti,ab. OR Yoga/ OR  Diet Therapy/ OR Diet/ OR Diet, Healthy/ OR Nutrition Therapy/ OR Fasting/ OR “Diet, Food, and Nutrition”/ OR Food Preferences/ OR ((health* OR unhealthy* OR poor) adj3 (eat* OR diet* OR food OR nutrition)).ti,ab. OR (diet* adj3 (intake OR consumption OR intervention* OR modif* OR chang*)).ti,ab. OR ((fruit* OR vegetable* OR salt OR sodium OR fat*) adj3 (intake OR consumption OR intervention* OR eat*)).ti,ab. OR  Screen Time/ OR ((computer OR digital media OR electronic device OR mobile device OR social media OR internet) adj3 (use* OR time)).ti,ab. OR (screen addiction OR television view* OR tv view* OR television watch* OR tv watch* OR watch* tv OR play* video game* OR work* on computer OR time spent on device*).ti,ab. OR Sedentary Behavior/ OR (sedentary adj3 (behavio?r* OR lifestyle OR life style OR time*)).ti,ab. OR (Physical inactivity OR prolonged sitting OR sitting time).ti,ab. OR  Tobacco Smoking/ OR Cigarette Smoking/ OR Smoking Cessation/ OR Smoking/ OR Cigar Smoking/ OR "Tobacco Use"/ OR "Tobacco Use Disorder"/ OR "Tobacco Use Cessation"/ OR Smokers/ OR Vaping/ OR (nicotine addict* OR quit smok*).ti,ab. OR  Alcohol Drinking/ OR Alcohol-Related Disorders/ OR Drinking Behavior/ OR (alcohol adj3 (abus* OR misus* OR intak* OR drink* OR use* OR consumpt* OR addict* OR withdrawal)).ti,ab. OR (drink* adj3 (alcohol OR habit* OR behavio?r*)).ti,ab. OR  Sleep/ OR Sleep Deprivation/ OR Sleep Hygiene/ OR Sleep Duration/ OR Sleep Quality/ OR Insomnia/ | Blood Glucose/ OR Glycemic Index/ OR Hyperglycemia/ OR (blood sugar OR glucometer OR glucose level* OR hemoglobin A1c OR HbA1c OR glycemic ind*).ti,ab. OR  Lipids/ OR Cholesterol, LDL/ OR Cholesterol/ OR Cholesterol, HDL/ OR Cholesterol, VLDL/ OR Hyperlipidemias/ OR Triglycerides/ OR (Blood lipid* OR lipid profile* OR total cholesterol).ti,ab. OR  Blood Pressure/ OR Hypertension/ OR (blood pressure OR BP).ti,ab. OR  Body Weight/ OR Waist Circumference/ OR Waist-Height Ratio/ OR Skinfold Thickness/ OR Body Mass Index/ OR Obesity/ OR Overweight/ OR (Weight OR BMI).ti,ab. OR (Waist OR hip) adj3 (circumference).ti,ab OR (Waist-to-height OR Waist-Height OR hip-to-height OR hip-height) adj3 (ratio).ti,ab | Randomized controlled trial |

**EMBASE Logic Grid Search**

Date of search: 04/04/2024

| **Digital interventions** | **Lifestyle** | **CVD risk factor** | **RCT** |
| --- | --- | --- | --- |
| 'telemedicine'/exp OR 'video consultation' OR 'telephone' OR 'teleconsultation' OR videoconferencing OR 'wearable computer'/exp OR 'video game' OR 'virtual reality' OR 'web-based intervention' OR 'mobile application'/exp OR 'mobile phone'/exp OR 'personal digital assistant' OR 'text messaging' OR (telemed*:ti,ab OR 'tele med*':ti,ab OR telehealth*:ti,ab OR 'tele health*':ti,ab OR 'internet based':ti,ab) AND intervent*:ti,ab OR ((ehealth*:ti,ab OR 'e health*':ti,ab OR electronic:ti,ab) AND health*:ti,ab OR mhealth*:ti,ab OR 'm health*':ti,ab OR mobile:ti,ab) AND health*:ti,ab OR 'telehealth'/exp OR (wearable* NEAR/3 (devic* OR technolog* OR sens*)):ti,ab OR ((digital* OR remote OR internet* OR online* OR 'on line*' OR virtual* OR 'web based*') NEAR/3 (coach* OR health* OR interven* OR program* OR therap*)):ti,ab OR 'social media' OR (health* NEAR/3 (chatbot* OR technolog* OR websit*)):ti,ab OR 'text messag*':ti,ab OR text*:ti,ab OR sms:ti,ab OR 'multi-media messag*':ti,ab OR 'multimedia messag*':ti,ab OR mms:ti,ab OR 'intsnat messag*':ti,ab OR 'picture messag*':ti,ab OR 'audio messag*':ti,ab OR gamification OR 'wii fit':ti,ab OR 'online forum*':ti,ab OR 'on-line forum*':ti,ab OR 'discussion board*':ti,ab | 'health behavior'/exp OR 'health promotion' OR 'health promotion through physical activity' OR 'risk reduction' OR 'behavior therapy' OR 'health education'/exp OR 'lifestyle' OR 'lifestyle modification' OR 'healthy lifestyle' OR ((lifestyle OR 'life style' OR behavio?r) NEAR/3 (chang* OR modif* OR program* OR therap* OR intervent*)):ti,ab OR 'health habit*':ti,ab OR 'unhealthy lifestyle*':ti,ab OR  exercise OR 'physical activity'/exp OR 'fitness' OR 'sport' OR 'walking' OR 'running' OR 'swimming' OR ((physical OR cardio* OR aerobic OR fitness OR endurance OR flexibility OR strength OR resistance) NEAR/3 (activit* OR train* OR exercise*)):ti,ab OR yoga OR 'diet therapy'/exp OR 'diet' OR 'dietary intake'/exp OR 'nutrition'/exp OR 'overnutrition'/exp OR 'healthy diet' OR OR 'unhealthy diet' OR 'fasting' OR 'food preference' OR ((health* OR unhealthy* OR poor) NEAR/3 (eat* OR diet* OR food OR nutrition)):ti,ab OR (diet* NEAR/3 (intake OR consumption OR intervention* OR modif* OR chang*)):ti,ab OR ((fruit* OR vegetable* OR salt OR sodium OR fat*) NEAR/3 (intake OR consumption OR intervention* OR eat*)):ti,ab OR  'screen time' OR ((computer OR 'digital media' OR 'electronic device' OR 'mobile device' OR 'social media' OR internet) NEAR/3 (use* OR time)):ti,ab OR 'screen addiction':ti,ab OR 'television view*':ti,ab OR 'tv view*':ti,ab OR 'television watch*':ti,ab OR 'tv watch*':ti,ab OR 'watch* tv':ti,ab OR 'play* video game*':ti,ab OR 'work* on computer':ti,ab OR 'time spent on device*':ti,ab OR  'sedentary lifestyle' OR (sedentary NEAR/3 (behavio?r* OR lifestyle OR 'life style' OR time*)):ti,ab OR 'physical inactivity':ti,ab OR 'prolonged sitting':ti,ab OR 'sitting time':ti,ab OR 'smoking' OR 'cigarette smoking' OR 'smoking cessation' OR 'cigar smoking' OR 'tobacco use' OR 'tobacco dependence' OR 'vaping' OR 'nicotine addict*':ti,ab OR 'quit smok*':ti,ab OR  'drinking behavior' OR 'alcoholism' OR (alcohol NEAR/3 (abus* OR misus* OR intak* OR drink* OR use* OR consumpt* OR addict* OR withdrawal)):ti,ab OR (drink* NEAR/3 (alcohol OR habit* OR behavio?r*)):ti,ab OR  'sleep' OR 'sleep quality' OR 'sleep time' OR 'sleep pattern' OR 'sleep disorder' OR 'sleep deprivation' OR 'sleep hygiene' OR ‘Insomnia’ | 'glucose blood level' OR 'blood glucose meter' OR 'glycemic index' OR 'hyperglycemia' OR 'hemoglobin a1c' OR 'blood sugar':ti,ab OR glucometer:ti,ab OR 'glucose level*':ti,ab OR hba1c:ti,ab OR 'glycemic ind*':ti,ab OR  'lipid' OR 'cholesterol blood level' OR 'high density lipoprotein cholesterol' OR 'low density lipoprotein cholesterol' OR 'very low density lipoprotein cholesterol' OR 'total cholesterol level' OR 'hyperlipidemia' OR 'triacylglycerol' OR 'blood lipid*':ti,ab OR 'lipid profile*':ti,ab OR 'total cholesterol':ti,ab OR  'blood pressure' OR 'hypertension' OR 'blood pressure':ti,ab OR ‘BP’:ti,ab OR  'body weight' OR 'body mass' OR 'obesity' OR 'waist circumference' OR 'waist to height ratio' OR 'skinfold thickness' OR weight:ti,ab OR 'body mass index':ti,ab OR bmi:ti,ab OR overweight:ti,ab OR ((waist OR hip) NEAR/3 circumference):ti,ab OR (('waist to height' OR 'waist height' OR 'hip to height' OR 'hip height') NEAR/3 ratio):ti,ab | Randomized controlled trial |

**Cochrane Central Register of Controlled Trials Search**

Date of search: 04/04/2024

| **Digital interventions** | **Lifestyle** | **CVD risk factor** | **RCT** |
| --- | --- | --- | --- |
| MeSH descriptor: [Telemedicine] OR [Telephone] OR [Videoconferencing] OR [Wearable Electronic Devices] OR [Video Games] OR [Virtual Reality] OR [Internet-Based Intervention] OR [Mobile Applications] OR [Cell Phone] OR [Smartphone] OR [Text Messaging] OR [Gamification] OR (telemed* OR tele-med* OR telehealth* OR tele-health*):ti,ab OR (ehealth* OR e-health* OR "electronic health*" OR mhealth* OR m-health* OR "mobile health*"):ti,ab OR (wearable* NEAR/3 (devic* OR technolog* OR sens*)):ti,ab OR ((digital* OR remote OR internet* OR online* OR on-line* OR "social media*" OR virtual* OR web-based*) NEAR/3 (coach* OR health* OR interven* OR program* OR therap*)):ti,ab OR (health* NEAR/3 (chatbot* OR technolog* OR websit*)):ti,ab OR ("text messag*" OR text* OR SMS OR "multi-media messag*" OR "multimedia messag*" OR MMS OR "intsnat messag*" OR "picture messag*" OR "audio messag*"):ti,ab OR (“wii fit” OR "online forum*" OR "on-line forum*" OR "discussion board*"):ti,ab | MeSH descriptor: [Health Behavior] OR [Health Promotion] OR [Risk Reduction Behavior] OR [Behavior Therapy] OR [Cognitive Behavioral Therapy] OR [Motivation] OR [Life Style] OR [Healthy Lifestyle] OR ((lifestyle OR "life style" OR behavio?r) NEAR/3 (chang* OR modif* OR program* OR therap* OR intervent*)):ti,ab OR ("health habit*" OR "unhealthy lifestyle*"):ti,ab OR [Exercise] OR [Exercise Therapy] OR [Physical Fitness] OR [Sports] OR [Walking] OR [Running] OR [Swimming] OR [Yoga] OR ((physical OR cardio* OR aerobic OR fitness OR endurance OR flexibility OR strength OR resistance) NEAR/3 (activit* OR train* OR exercise*)):ti,ab OR  [Diet] OR [Diet Therapy] OR [Diet, Healthy] OR [Nutrition Therapy] OR [Fasting] OR [Food Preferences] OR ((health* OR unhealthy* OR poor) NEAR/3 (eat* OR diet* OR food OR nutrition)):ti,ab OR (diet* NEAR/3 (intake OR consumption OR intervention* OR modif* OR chang*)):ti,ab OR ((fruit* OR vegetable* OR salt OR sodium OR fat*) NEAR/3 (intake OR consumption OR intervention* OR eat*)):ti,ab OR  [Screen Time] OR ((computer OR "digital media" OR "electronic device" OR "mobile device" OR "social media" OR internet) NEAR/3 (use* OR time)):ti,ab OR ("screen addiction" OR "television view*" OR "tv view*" OR "television watch*" OR "tv watch*" OR "watch* tv" OR "play* video game*" OR "work* on computer" OR "time spent on device*"):ti,ab OR [Sedentary Behavior] OR (sedentary NEAR/3 (behavio?r* OR lifestyle OR "life style" OR time*)):ti,ab OR ("Physical inactivity" OR "prolonged sitting" OR "sitting time"):ti,ab OR  [Smoking] OR [Tobacco Smoking] OR [Cigarette Smoking] OR [Smoking Cessation] OR [Cigar Smoking] OR [Tobacco Use] OR [Tobacco Use Cessation] OR [Tobacco Use Disorder] OR [Smokers] OR [Vaping] OR  [Alcohol Drinking] OR [Alcohol-Related Disorders] OR [Drinking Behavior] OR (alcohol NEAR/3 (abus* OR misus* OR intak* OR drink* OR use* OR consumpt* OR addict* OR withdrawal)):ti,ab OR(drink* NEAR/3 (alcohol OR habit* OR behavio?r*)):ti,ab OR  [Sleep] OR [Sleep Deprivation] OR [Sleep Hygiene] OR [Sleep Duration] OR [Sleep Quality] OR [Sleep Initiation and Maintenance Disorders] | MeSH descriptor: [Blood Glucose] OR [Glycemic Index] OR [Hyperglycemia] OR [Glycated Hemoglobin] OR ("blood sugar" OR glucometer OR "glucose level*" OR "hemoglobin A1c" OR HbA1c OR "glycemic ind*"):ti,ab OR  [Lipids] OR [Cholesterol, LDL] OR [Cholesterol, VLDL] OR [Cholesterol, HDL] OR [Cholesterol] OR [Hyperlipidemias] OR [Triglycerides] OR ("Blood lipid*" OR "lipid profile*" OR "total cholesterol"):ti,ab OR  [Blood Pressure] OR [Hypertension] OR ("blood pressure"):ti,ab OR ‘BP’:ti,ab OR  [Body Weight] OR [Body Mass Index] OR [Obesity] OR [Overweight] OR [Waist Circumference] OR [Waist-Height Ratio] OR [Skinfold Thickness] OR (Weight OR BMI):ti,ab OR ((waist OR hip) NEAR/3 circumference):ti,ab OR (('waist to height' OR 'waist height' OR 'hip to height' OR 'hip height') NEAR/3 ratio):ti,ab | Randomized controlled trial |

**CINAHL Search**

Date of search: 04/04/2024

| **Digital interventions** | **Lifestyle** | **CVD risk factor** | **RCT** |
| --- | --- | --- | --- |
| telemedicine OR telehealth OR ehealth or e-health OR electronic health OR mhealth OR mobile health OR m-health OR mobile app OR mobile application OR smartphone application OR app OR apps OR telephone consultation OR remote consultation OR telephone review OR telephone consultation OR video conferencing OR video conference OR videoconferencing OR videoconference OR wearable technology OR wearable devices OR wearable sensors OR wearable data OR smartwatch OR fitness tracker OR video games OR computer games OR gaming OR online games OR internet games OR online gaming OR gamification OR virtual reality OR vr OR augmented reality OR social media OR internet-based interventions OR web-based interventions OR internet based therapy OR online intervention OR internet intervention OR web intervention OR digital intervention OR cell phones OR mobile phones OR smart phones OR mobile devices OR text messaging OR texting OR sms messaging OR ((digital* OR remote OR internet* OR onlin* OR on-lin* OR "social media*" OR virtual OR web-based) n3 (coach* OR health* OR interven* OR progra?m* OR therap* OR conference*)) OR (health* n3 (chatbot* OR technolog* OR websit*)) OR "text messag*" OR text* OR sms OR "multi media messag*" OR "multimedia messag*" OR mms OR "intsnat messag*" OR "picture messag*" OR "audio messag*" OR "wii fit" OR "online forum" OR "on-line forum" OR "discussion board*" | cognitive behavioral therapy OR cbt OR cognitive behavioural therapy OR behavior therapy OR behavior modification OR behavior intervention OR risk reduction OR health promotion OR health education OR motivation OR lifestyle OR healthy lifestyle OR health practices OR health behaviors OR lifestyle modification OR lifestyle change OR lifestyle intervention OR  exercise OR physical activity OR fitness OR aerobic training OR strength training OR cardiovascular training OR cardio OR walking OR exercise therapy OR exercise intervention OR neuromuscular training OR sports OR swimming OR running OR jogging OR run OR jog OR walking program OR walking intervention OR yoga OR yoga therapy OR yoga exercise OR yoga practice OR yoga intervention OR ((physical OR cardio* OR aerobic OR fitness OR endurance OR fexibilit* OR strength OR resistance) n3 (activit* OR train* OR exercise*)) OR  diet OR nutrition OR food habit OR eating habit OR food OR dietary intake OR diet therapy OR nutrition therapy OR food preferences OR fasting OR intermittent fasting OR time-restricted feeding OR time-restricted fasting OR  ((health* OR unhealth* OR poor) n3 (eat* OR diet* OR food OR nutrition)) OR (diet n3 (intak* OR consumption* OR interventio* OR modif* OR chang*)) OR ((fruit* OR vegetabl* OR salt OR sodium OR fat*) n3 (intake OR consumption OR intervention OR eat*)) OR  screen-time OR screen time OR screen exposure OR screen use OR digital media use OR ((computer OR "digital media" OR "electronic devic*" OR "mobile devic*" OR "social media" OR internet) n3 (use OR time)) OR "screen addiction" OR "television view*" OR "tv view*" OR "television watch*" OR "tv watch*" OR watch* OR "watch* tv" OR "play* video gam*" OR work* OR computer OR "time spent on devic*" OR  sedentary behavior OR physical inactivity OR (sedentary n3 (behavio?r OR lifestyle OR "life style" OR tim*)) OR "physical inactivit*" OR "prolonged sit*" OR "sit* time" OR  Tobacco Smoking OR Cigarette Smoking OR Smoking OR Cigar Smoking OR smoking cessation OR smoking cessation interventions OR quit smoking OR stop smoking OR smoking cessation programs OR tobacco use OR vaping OR electronic cigarettes OR e-cigarettes OR nicotine addiction OR  alcohol drinking OR alcohol drinking habit OR drinking behavior OR alcoholism OR alcohol dependence OR alcohol abuse OR alcoholic OR alcohol addiction OR alcohol use disorder OR alcohol misuse OR aud OR (alcohol n3 (abus* OR misus* OR intak* OR drink* OR us* OR consump* OR addict* OR withdrawal)) OR (drink* n3 (alcohol OR habit* OR behavio?r)) OR  sleep deprivation OR sleep disturbance OR reduced sleep OR sleep loss OR lack of sleep OR sleep quality OR sleep OR sleep hygiene OR insomnia | blood glucose levels OR blood sugar OR blood glucose OR glycemic control OR glycemic index OR glycemic load OR hyperglycemia OR glucometer OR hemoglobin a1c OR hba1c OR glycosylated hemoglobin OR  lipids OR cholesterol OR triglycerides OR cholesterol ldl OR cholesterol, hdl OR cholesterol, vldl OR hyperlipidemias OR total cholesterol OR  blood pressure OR bp OR hypertension OR hypertensive OR htn OR elevated blood pressure OR  body weight OR waist circumference OR waist height ratio OR skinfold thickness OR body mass index OR bmi OR obesity OR overweight OR (("waist-to-height" OR "waist-height" OR "hip-to-height" OR "hip-height”) n3 (ratio)) | Randomized controlled trial |

**PsycINFO Search**

Date of search: 04/04/2024

| **Digital interventions** | **Lifestyle** | **CVD risk factor** | **RCT** |
| --- | --- | --- | --- |
| telemedicine OR telehealth OR ehealth or e-health OR electronic health OR mhealth OR mobile health OR m-health OR mobile app OR mobile application OR smartphone application OR app OR apps OR telephone consultation OR remote consultation OR telephone review OR telephone consultation OR video conferencing OR video conference OR videoconferencing OR videoconference OR wearable technology OR wearable devices OR wearable sensors OR wearable data OR smartwatch OR fitness tracker OR video games OR computer games OR gaming OR online games OR internet games OR online gaming OR gamification OR virtual reality OR vr OR augmented reality OR social media OR internet-based interventions OR web-based interventions OR internet based therapy OR online intervention OR internet intervention OR web intervention OR digital intervention OR cell phones OR mobile phones OR smart phones OR mobile devices OR text messaging OR texting OR sms messaging OR ((digital* OR remote OR internet* OR onlin* OR on-lin* OR "social media*" OR virtual OR web-based) n3 (coach* OR health* OR interven* OR progra?m* OR therap* OR conference*)) OR (health* n3 (chatbot* OR technolog* OR websit*)) OR "text messag*" OR text* OR sms OR "multi media messag*" OR "multimedia messag*" OR mms OR "intsnat messag*" OR "picture messag*" OR "audio messag*" OR "wii fit" OR "online forum" OR "on-line forum" OR "discussion board*" | cognitive behavioral therapy OR cbt OR cognitive behavioural therapy OR behavior therapy OR behavior modification OR behavior intervention OR risk reduction OR health promotion OR health education OR motivation OR lifestyle OR healthy lifestyle OR health practices OR health behaviors OR lifestyle modification OR lifestyle change OR lifestyle intervention OR  exercise OR physical activity OR fitness OR aerobic training OR strength training OR cardiovascular training OR cardio OR walking OR exercise therapy OR exercise intervention OR neuromuscular training OR sports OR swimming OR running OR jogging OR run OR jog OR walking program OR walking intervention OR yoga OR yoga therapy OR yoga exercise OR yoga practice OR yoga intervention OR ((physical OR cardio* OR aerobic OR fitness OR endurance OR fexibilit* OR strength OR resistance) n3 (activit* OR train* OR exercise*)) OR  diet OR nutrition OR food habit OR eating habit OR food OR dietary intake OR diet therapy OR nutrition therapy OR food preferences OR fasting OR intermittent fasting OR time-restricted feeding OR time-restricted fasting OR  ((health* OR unhealth* OR poor) n3 (eat* OR diet* OR food OR nutrition)) OR (diet n3 (intak* OR consumption* OR interventio* OR modif* OR chang*))  OR ((fruit* OR vegetabl* OR salt OR sodium OR fat*) n3 (intake OR consumption OR intervention OR eat*)) OR  screen-time OR screen time OR screen exposure OR screen use OR digital media use OR ((computer OR "digital media" OR "electronic devic*" OR "mobile devic*" OR "social media" OR internet) n3 (use OR time)) OR "screen addiction" OR "television view*" OR "tv view*" OR "television watch*" OR "tv watch*" OR watch* OR "watch* tv" OR "play* video gam*" OR work* OR computer OR "time spent on devic*" OR sedentary behavior OR physical inactivity OR (sedentary n3 (behavio?r OR lifestyle OR "life style" OR tim*)) OR "physical inactivit*" OR "prolonged sit*" OR "sit* time" OR  Tobacco Smoking OR Cigarette Smoking OR Smoking OR Cigar Smoking OR smoking cessation OR smoking cessation interventions OR quit smoking OR stop smoking OR smoking cessation programs OR tobacco use OR vaping OR electronic cigarettes OR e-cigarettes OR nicotine addiction OR  alcohol drinking OR alcohol drinking habit OR drinking behavior OR alcoholism OR alcohol dependence OR alcohol abuse OR alcoholic OR alcohol addiction OR alcohol use disorder OR alcohol misuse OR aud OR (alcohol n3 (abus* OR misus* OR intak* OR drink* OR us* OR consump* OR addict* OR withdrawal)) OR (drink* n3 (alcohol OR habit* OR behavio?r)) OR  sleep deprivation OR sleep disturbance OR reduced sleep OR sleep loss OR lack of sleep OR sleep quality OR sleep OR sleep hygiene OR insomnia | blood glucose levels OR blood sugar OR blood glucose OR glycemic control OR glycemic index OR glycemic load OR hyperglycemia OR glucometer OR hemoglobin a1c OR hba1c OR glycosylated hemoglobin OR  lipids OR cholesterol OR triglycerides OR cholesterol ldl OR cholesterol, hdl OR cholesterol, vldl OR hyperlipidemias OR total cholesterol OR  blood pressure OR bp OR hypertension OR hypertensive OR htn OR elevated blood pressure OR  body weight OR waist circumference OR waist height ratio OR skinfold thickness OR body mass index OR bmi OR obesity OR overweight OR (("waist-to-height" OR "waist-height" OR "hip-to-height" OR "hip-height”) n3 (ratio)) | Randomized controlled trial |

**SPORTDiscus with Full Text Search**

Date of search: 04/04/2024

| **Digital interventions** | **Lifestyle** | **CVD risk factor** | **RCT** |
| --- | --- | --- | --- |
| telemedicine OR telehealth OR ehealth or e-health OR electronic health OR mhealth OR mobile health OR m-health OR mobile app OR mobile application OR smartphone application OR app OR apps OR telephone consultation OR remote consultation OR telephone review OR telephone consultation OR video conferencing OR video conference OR videoconferencing OR videoconference OR wearable technology OR wearable devices OR wearable sensors OR wearable data OR smartwatch OR fitness tracker OR video games OR computer games OR gaming OR online games OR internet games OR online gaming OR gamification OR virtual reality OR vr OR augmented reality OR social media OR internet-based interventions OR web-based interventions OR internet based therapy OR online intervention OR internet intervention OR web intervention OR digital intervention OR cell phones OR mobile phones OR smart phones OR mobile devices OR text messaging OR texting OR sms messaging OR ((digital* OR remote OR internet* OR onlin* OR on-lin* OR "social media*" OR virtual OR web-based) n3 (coach* OR health* OR interven* OR progra?m* OR therap* OR conference*)) OR (health* n3 (chatbot* OR technolog* OR websit*)) OR "text messag*" OR text* OR sms OR "multi media messag*" OR "multimedia messag*" OR mms OR "intsnat messag*" OR "picture messag*" OR "audio messag*" OR "wii fit" OR "online forum" OR "on-line forum" OR "discussion board*" | cognitive behavioral therapy OR cbt OR cognitive behavioural therapy OR behavior therapy OR behavior modification OR behavior intervention OR risk reduction OR health promotion OR health education OR motivation OR lifestyle OR healthy lifestyle OR health practices OR health behaviors OR lifestyle modification OR lifestyle change OR lifestyle intervention OR  exercise OR physical activity OR fitness OR aerobic training OR strength training OR cardiovascular training OR cardio OR walking OR exercise therapy OR exercise intervention OR neuromuscular training OR sports OR swimming OR running OR jogging OR run OR jog OR walking program OR walking intervention OR yoga OR yoga therapy OR yoga exercise OR yoga practice OR yoga intervention OR ((physical OR cardio* OR aerobic OR fitness OR endurance OR fexibilit* OR strength OR resistance) n3 (activit* OR train* OR exercise*)) OR  diet OR nutrition OR food habit OR eating habit OR food OR dietary intake OR diet therapy OR nutrition therapy OR food preferences OR fasting OR intermittent fasting OR time-restricted feeding OR time-restricted fasting OR  ((health* OR unhealth* OR poor) n3 (eat* OR diet* OR food OR nutrition)) OR (diet n3 (intak* OR consumption* OR interventio* OR modif* OR chang*)) OR ((fruit* OR vegetabl* OR salt OR sodium OR fat*) n3 (intake OR consumption OR intervention OR eat*)) OR  screen-time OR screen time OR screen exposure OR screen use OR digital media use OR ((computer OR "digital media" OR "electronic devic*" OR "mobile devic*" OR "social media" OR internet) n3 (use OR time)) OR "screen addiction" OR "television view*" OR "tv view*" OR "television watch*" OR "tv watch*" OR watch* OR "watch* tv" OR "play* video gam*" OR work* OR computer OR "time spent on devic*" OR  sedentary behavior OR physical inactivity OR (sedentary n3 (behavio?r OR lifestyle OR "life style" OR tim*)) OR "physical inactivit*" OR "prolonged sit*" OR "sit* time" OR  Tobacco Smoking OR Cigarette Smoking OR Smoking OR Cigar Smoking OR smoking cessation OR smoking cessation interventions OR quit smoking OR stop smoking OR smoking cessation programs OR tobacco use OR vaping OR electronic cigarettes OR e-cigarettes OR nicotine addiction OR  alcohol drinking OR alcohol drinking habit OR drinking behavior OR alcoholism OR alcohol dependence OR alcohol abuse OR alcoholic OR alcohol addiction OR alcohol use disorder OR alcohol misuse OR aud OR (alcohol n3 (abus* OR misus* OR intak* OR drink* OR use OR consump* OR addict* OR withdrawal)) OR (drink* n3 (alcohol OR habit* OR behavio?r)) OR  sleep deprivation OR sleep disturbance OR reduced sleep OR sleep loss OR lack of sleep OR sleep quality OR sleep OR sleep hygiene OR insomnia | blood glucose levels OR blood sugar OR blood glucose OR glycemic control OR glycemic index OR glycemic load OR hyperglycemia OR glucometer OR hemoglobin a1c OR hba1c OR glycosylated hemoglobin OR  lipids OR cholesterol OR triglycerides OR cholesterol ldl OR cholesterol, hdl OR cholesterol, vldl OR hyperlipidemias OR total cholesterol OR  blood pressure OR bp OR hypertension OR hypertensive OR htn OR elevated blood pressure OR  body weight OR waist circumference OR waist height ratio OR skinfold thickness OR body mass index OR bmi OR obesity OR overweight OR (("waist-to-height" OR "waist-height" OR "hip-to-height" OR "hip-height”) n3 (ratio)) | Randomized controlled trial |

**Web of Science Search**

Date of search: 04/04/2024

| (((TS=(telemedicine OR telehealth OR ehealth or e-health OR electronic health OR mhealth OR mobile health OR m-health OR mobile app OR mobile application OR smartphone application OR app OR apps OR telephone consultation OR remote consultation OR telephone review OR telephone consultation OR video conferencing OR video conference OR videoconferencing OR videoconference OR wearable technology OR wearable devices OR wearable sensors OR wearable data OR smartwatch OR fitness tracker OR video games OR computer games OR gaming OR online games OR internet games OR online gaming OR gamification OR virtual reality OR vr OR augmented reality OR social media OR internet-based interventions OR web-based interventions OR internet based therapy OR online intervention OR internet intervention OR web intervention OR digital intervention OR cell phones OR mobile phones OR smart phones OR mobile devices OR text messaging OR texting OR sms messaging OR ((digital* OR remote OR internet* OR onlin* OR on-lin* OR "social media*" OR virtual OR web-based) n3 (coach* OR health* OR interven* OR progra?m* OR therap* OR conference*)) OR (health* n3 (chatbot* OR technolog* OR websit*)) OR "text messag*" OR text* OR sms OR "multi media messag*" OR "multimedia messag*" OR mms OR "intsnat messag*" OR "picture messag*" OR "audio messag*" OR "wii fit" OR "online forum" OR "on-line forum" OR "discussion board*"))  AND  TS=(cognitive behavioral therapy OR cbt OR cognitive behavioural therapy OR behavior therapy OR behavior modification OR behavior intervention OR risk reduction OR health promotion OR health education OR motivation OR lifestyle OR healthy lifestyle OR health practices OR health behaviors OR lifestyle modification OR lifestyle change OR lifestyle intervention OR exercise OR physical activity OR fitness OR aerobic training OR strength training OR cardiovascular training OR cardio OR walking OR exercise therapy OR exercise intervention OR neuromuscular training OR sports OR swimming OR running OR jogging OR run OR jog OR walking program OR walking intervention OR yoga OR yoga therapy OR yoga exercise OR yoga practice OR yoga intervention OR ((physical OR cardio* OR aerobic OR fitness OR endurance OR fexibilit* OR strength OR resistance) n3 (activit* OR train* OR exercise*)) OR diet OR nutrition OR food habit OR eating habit OR food OR dietary intake OR diet therapy OR nutrition therapy OR food preferences OR fasting OR intermittent fasting OR time-restricted feeding OR time-restricted fasting OR ((health* OR unhealth* OR poor) n3 (eat* OR diet* OR food OR nutrition)) OR (diet n3 (intak* OR consumption* OR interventio* OR modif* OR chang*)) OR ((fruit* OR vegetabl* OR salt OR sodium OR fat*) n3 (intake OR consumption OR intervention OR eat*)) OR screen-time OR screen time OR screen exposure OR screen use OR digital media use OR ((computer OR "digital media" OR "electronic devic*" OR "mobile devic*" OR "social media" OR internet) n3 (use OR time)) OR "screen addiction" OR "television view*" OR "tv view*" OR "television watch*" OR "tv watch*" OR watch* OR "watch* tv" OR "play* video gam*" OR work* OR computer OR "time spent on devic*" OR sedentary behavior OR physical inactivity OR (sedentary n3 (behavio?r OR lifestyle OR "life style" OR tim*)) OR "physical inactivit*" OR "prolonged sit*" OR "sit* time" OR Tobacco Smoking OR Cigarette Smoking OR Smoking OR Cigar Smoking OR smoking cessation OR smoking cessation interventions OR quit smoking OR stop smoking OR smoking cessation programs OR tobacco use OR vaping OR electronic cigarettes OR e-cigarettes OR nicotine addiction OR alcohol drinking OR alcohol drinking habit OR drinking behavior OR alcoholism OR alcohol dependence OR alcohol abuse OR alcoholic OR alcohol addiction OR alcohol use disorder OR alcohol misuse OR aud OR (alcohol n3 (abus* OR misus* OR intak* OR drink* OR use OR consump* OR addict* OR withdrawal)) OR (drink* n3 (alcohol OR habit* OR behavio?r)) OR sleep deprivation OR sleep disturbance OR reduced sleep OR sleep loss OR lack of sleep OR sleep quality OR sleep OR sleep hygiene OR insomnia))  AND  TS=(blood glucose levels OR blood sugar OR blood glucose OR glycemic control OR glycemic index OR glycemic load OR hyperglycemia OR glucometer OR hemoglobin a1c OR hba1c OR glycosylated hemoglobin OR lipids OR cholesterol OR triglycerides OR cholesterol ldl OR cholesterol, hdl OR cholesterol, vldl OR hyperlipidemias OR total cholesterol OR blood pressure OR bp OR hypertension OR hypertensive OR htn OR elevated blood pressure OR body weight OR waist circumference OR waist height ratio OR skinfold thickness OR body mass index OR bmi OR obesity OR overweight OR (("waist-to-height" OR "waist-height" OR "hip-to-height" OR "hip-height”) n3 (ratio))))  AND  ALL=(randomized controlled trials OR rct OR randomised control trials OR clinical trial) |
| --- |


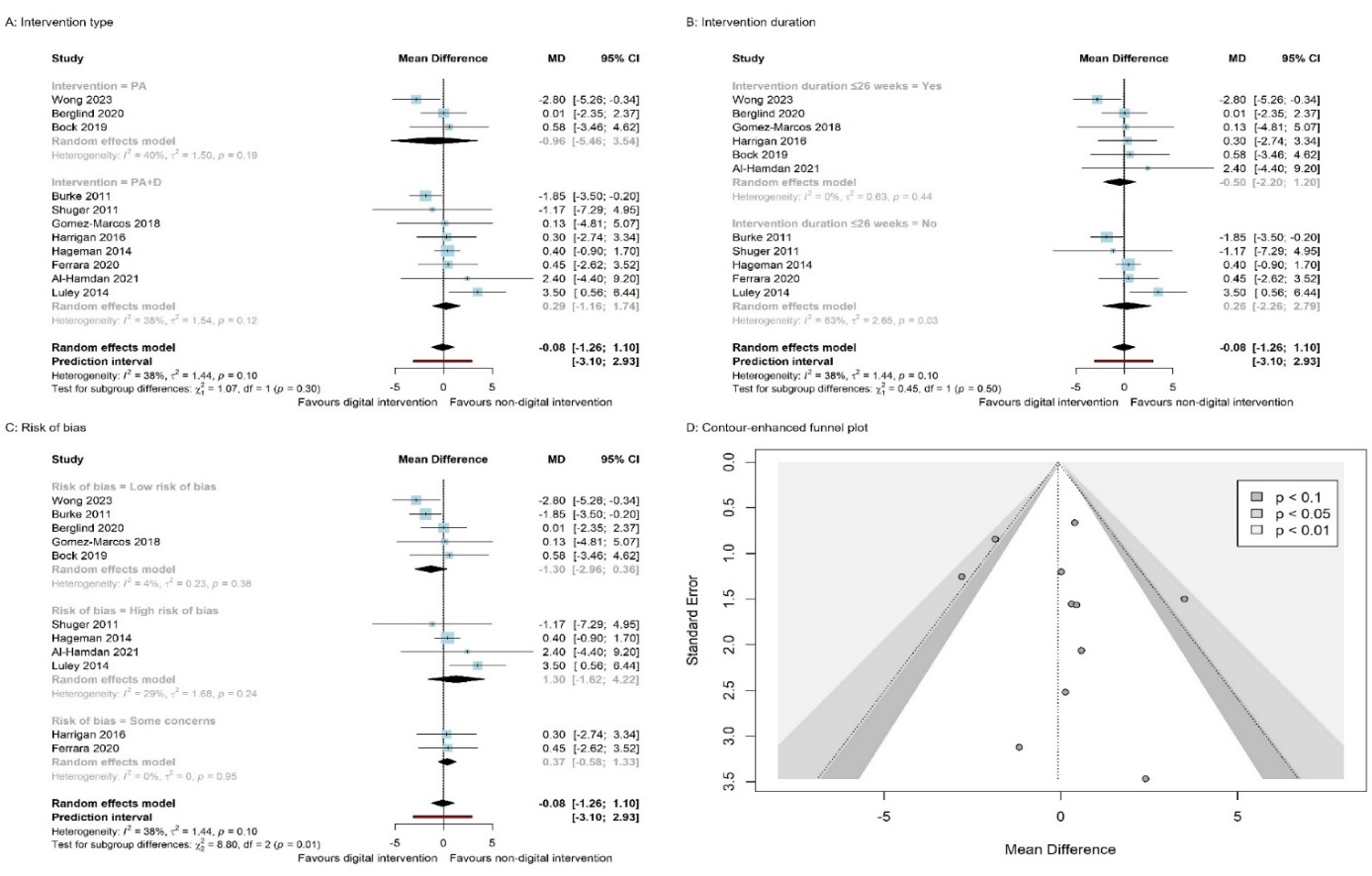


Supplementary Material 2: Waist circumference panel plots (A – C: Subgroup analysis forest plots and D: funnel plot)

#
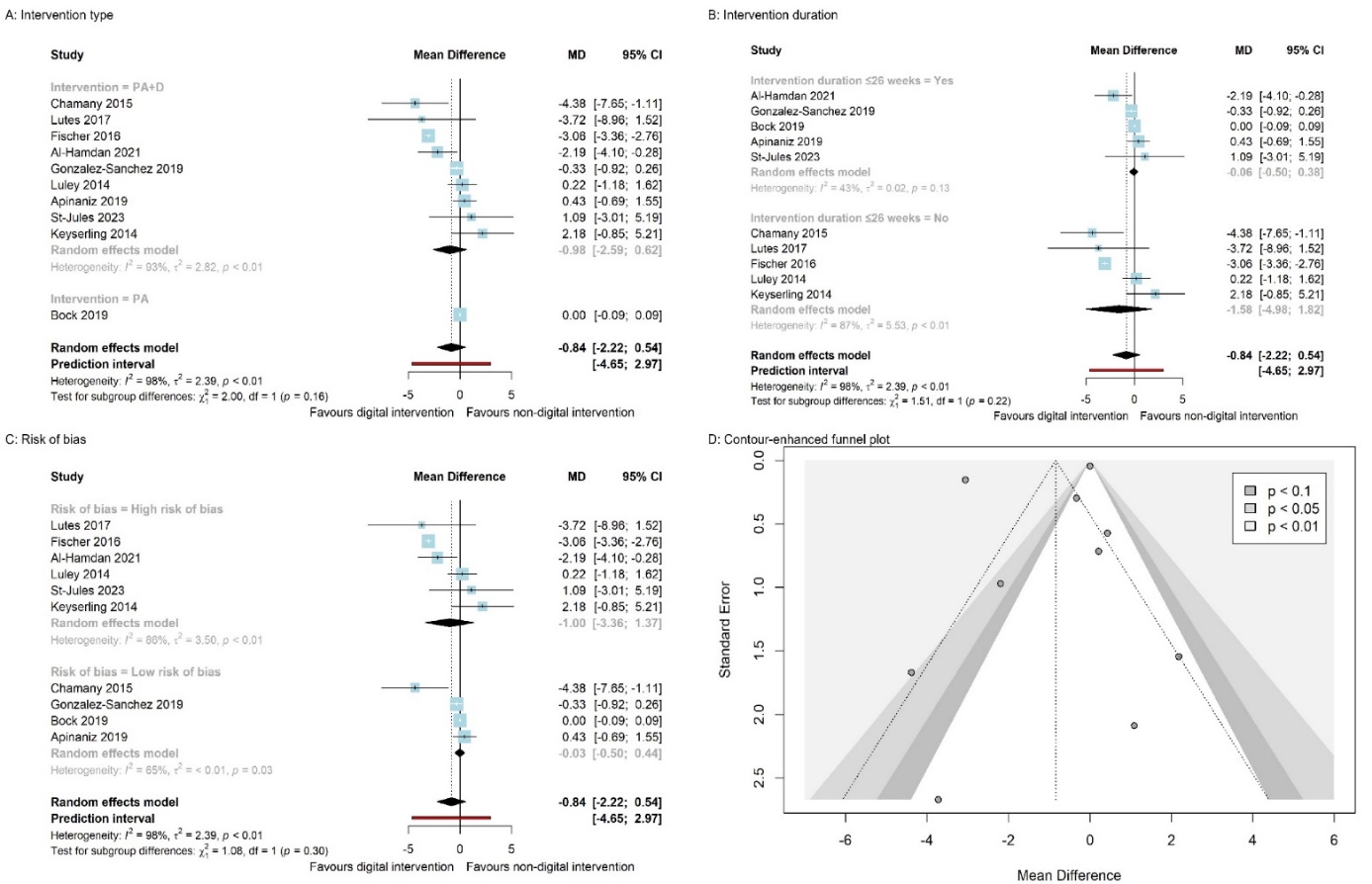


# Supplementary Material 3: HbA1c panel plots (A – C: Subgroup analysis forest plots and D: funnel plot)


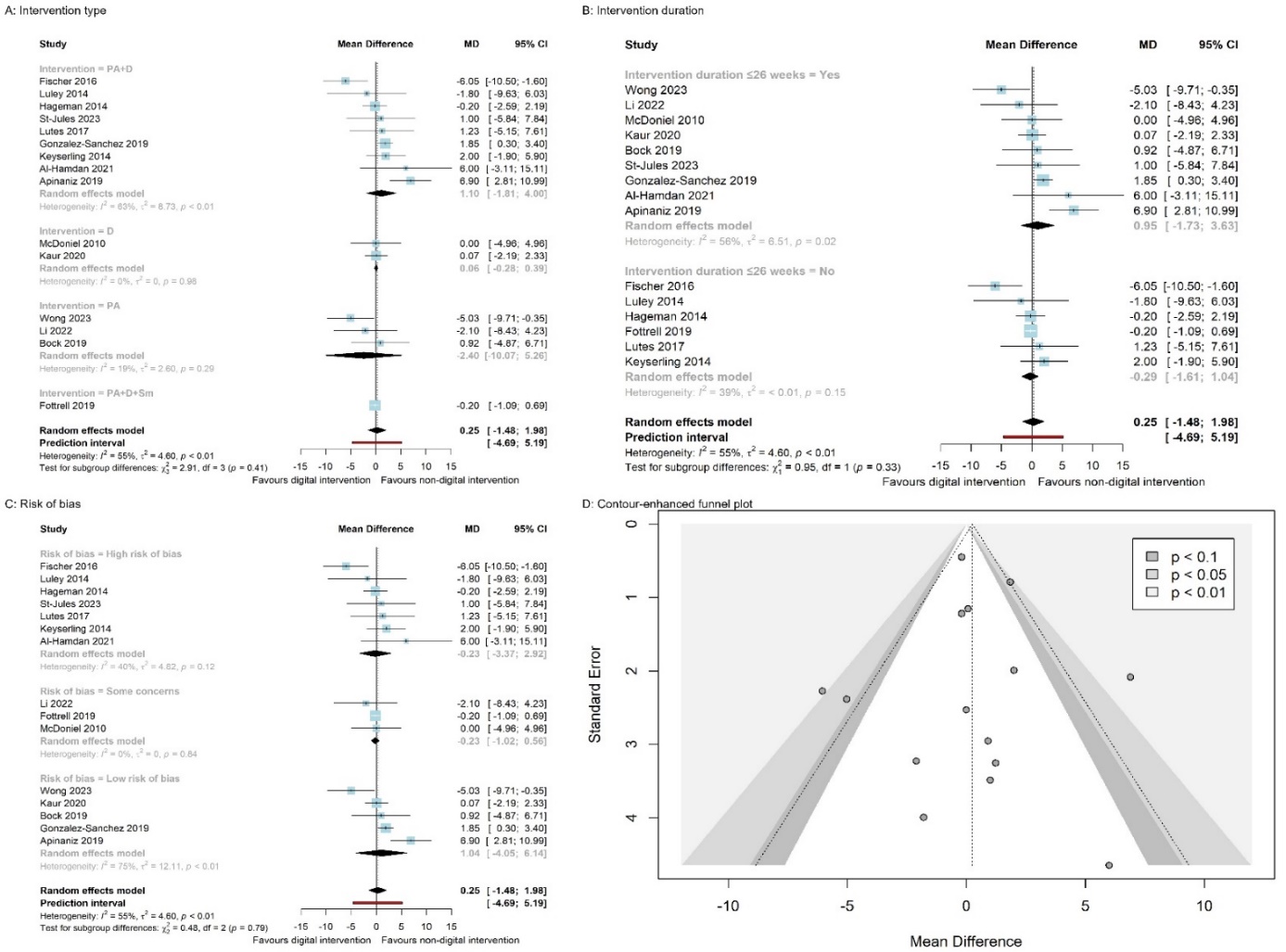


Supplementary Material 4: Systolic blood pressure panel plots (A – C: Subgroup analysis forest plots and D: funnel plot)

#
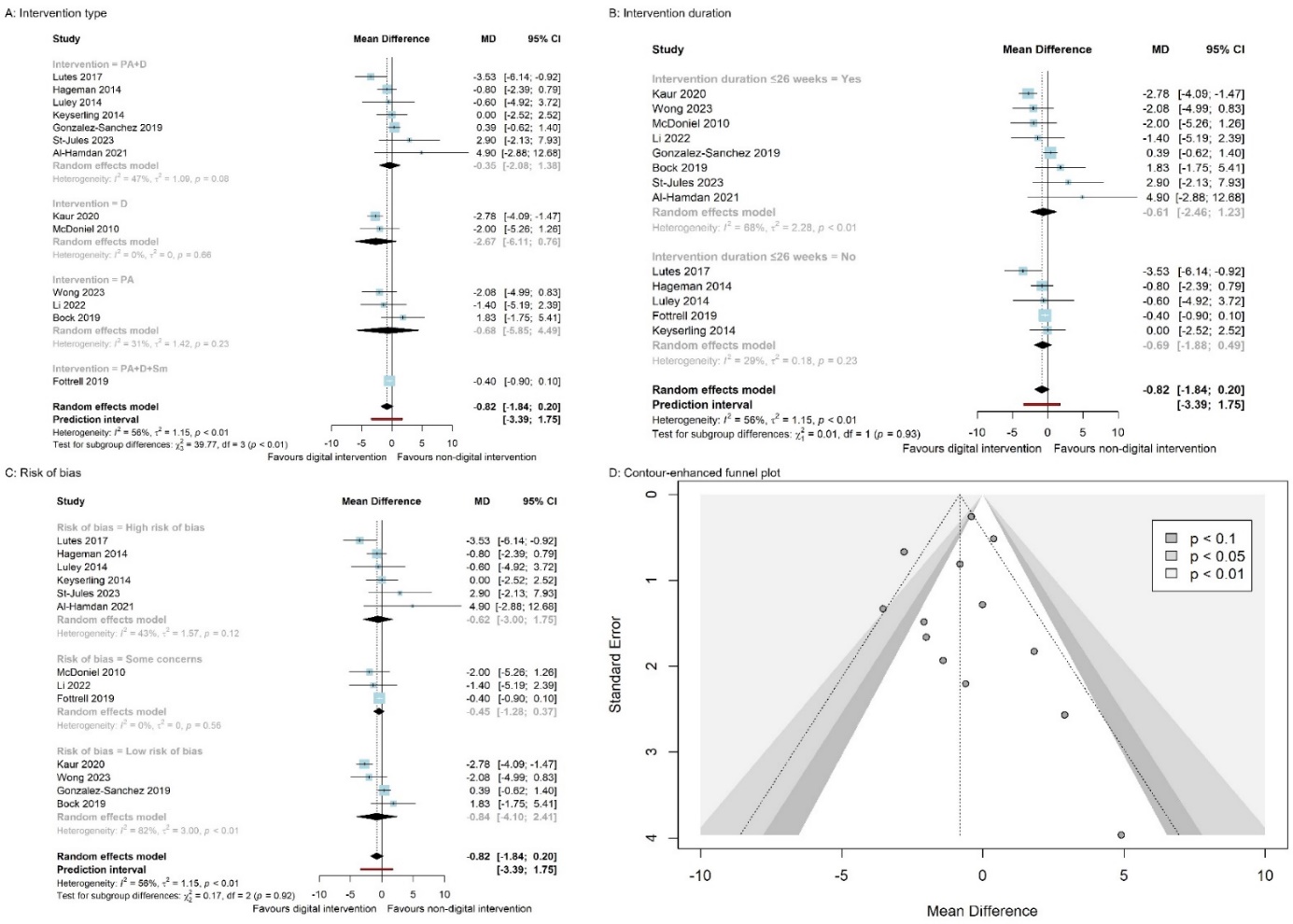


# Supplementary Material 5: Diastolic blood pressure panel plots (A – C: Subgroup analysis forest plots and D: funnel plot)


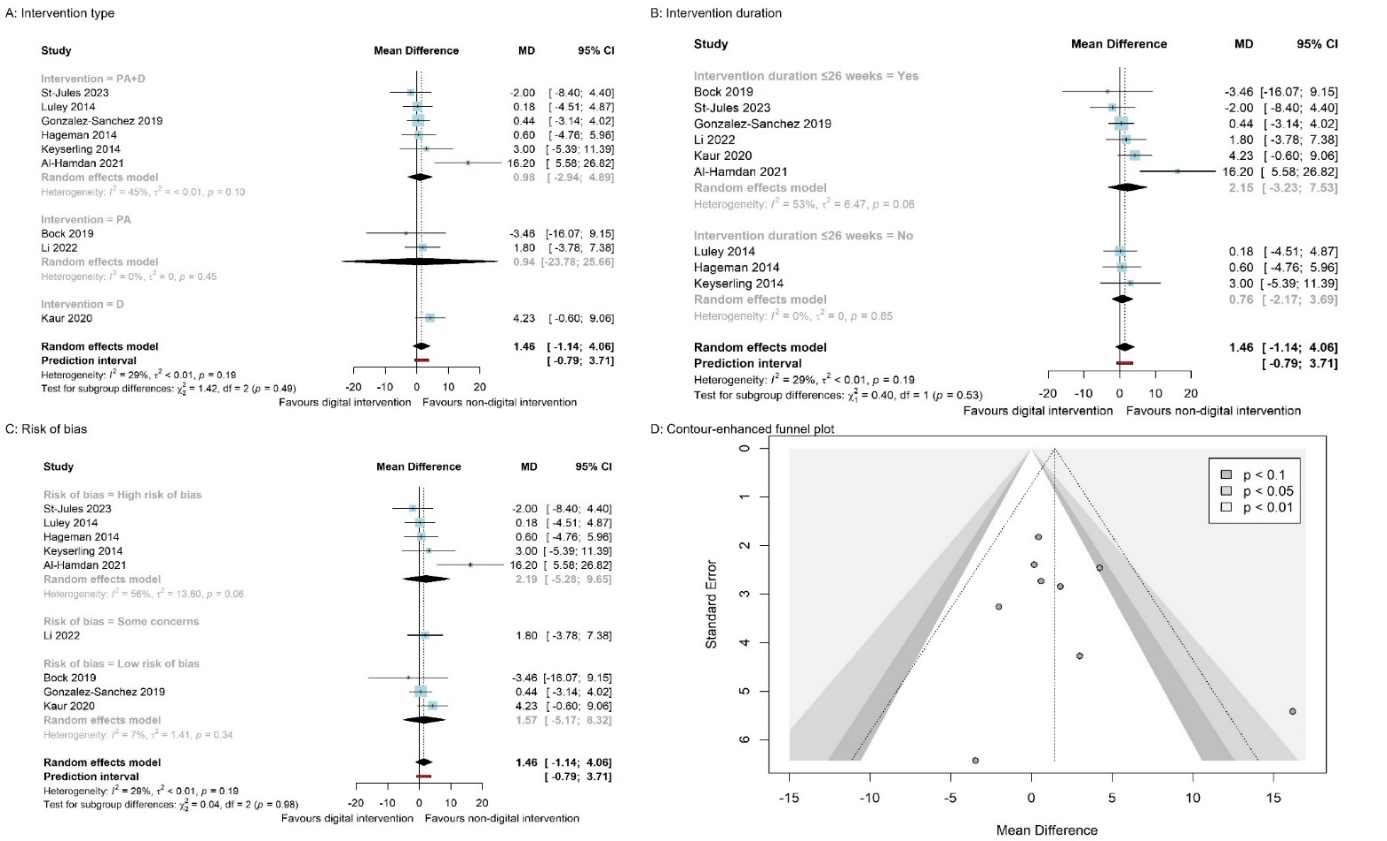


Supplementary Material 6: LDL cholesterol panel plots (A – C: Subgroup analysis forest plots and D: funnel plot)


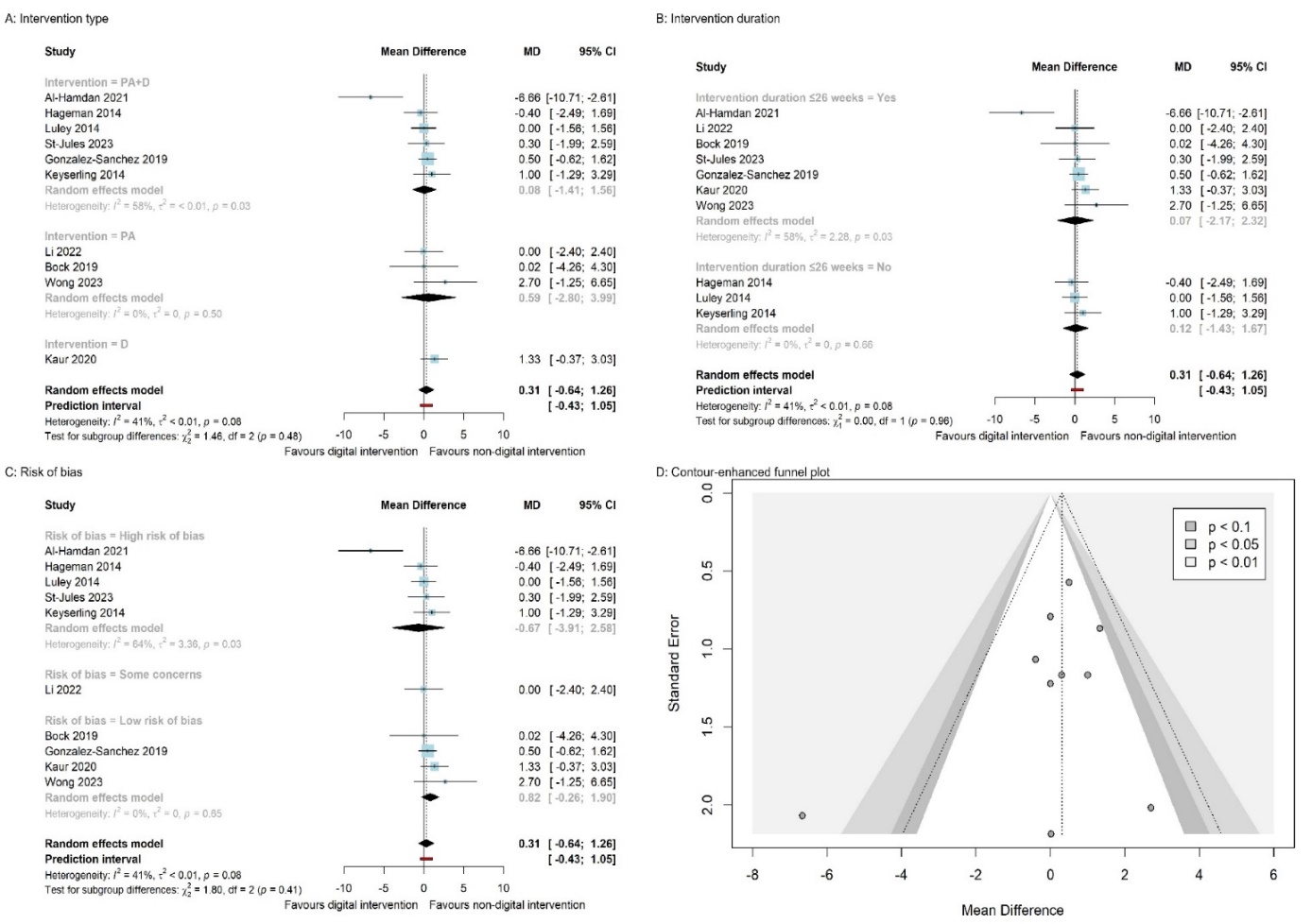
Supplementary Material 7: HDL cholesterol panel plots (A – C: Subgroup analysis forest plots and D: funnel plot)

#
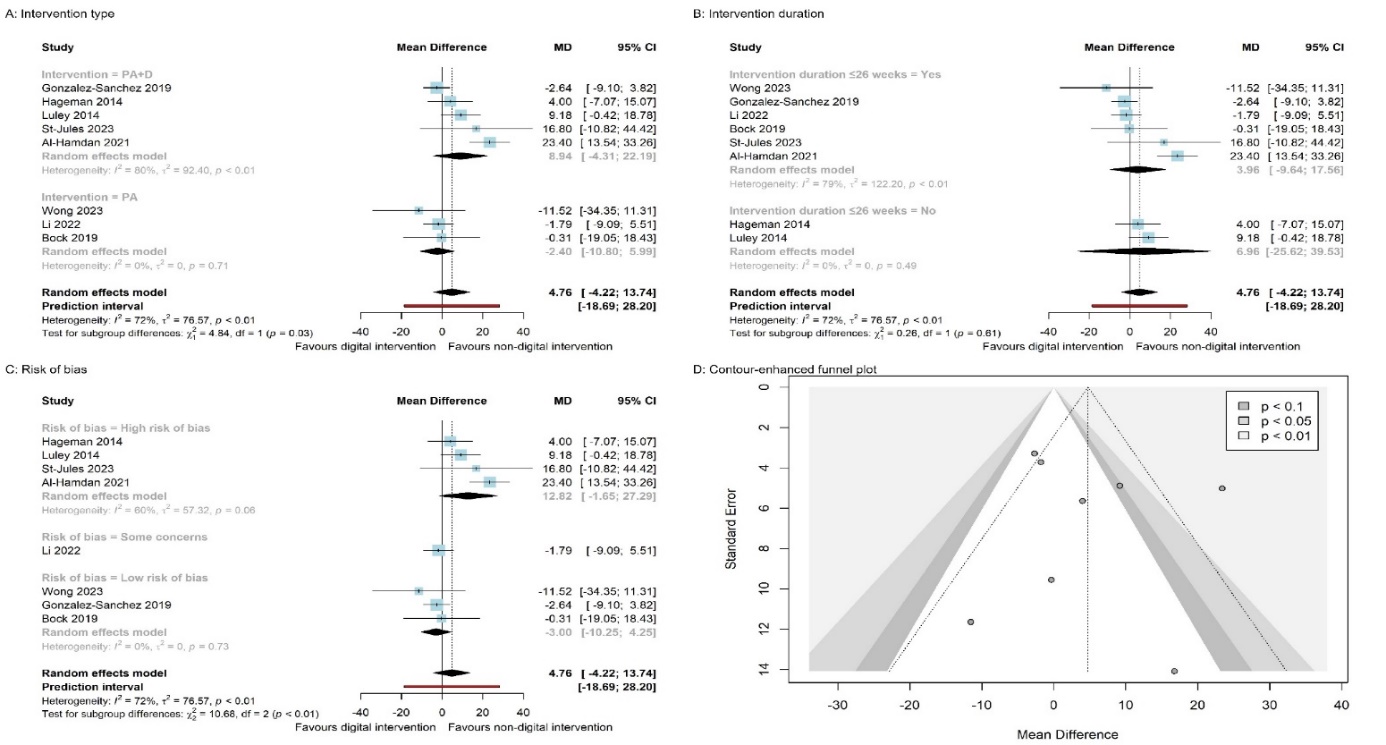


# Supplementary Material 8: Triglycerides panel plots (A – C: Subgroup analysis forest plots and D: funnel plot)
